# Notoginsenoside R1 Improves Cerebral Ischemia/Reperfusion Injury by Promoting Neurogenesis via the BDNF/Akt/CREB Pathway

**DOI:** 10.3389/fphar.2021.615998

**Published:** 2021-05-07

**Authors:** Ting Zhu, Lei Wang, Weijie Xie, Xiangbao Meng, Yicheng Feng, Guibo Sun, Xiaobo Sun

**Affiliations:** ^1^ Beijing Key Laboratory of Innovative Drug Discovery of Traditional Chinese Medicine (Natural Medicine) and Translational Medicine, Institute of Medicinal Plant Development, Peking Union Medical College and Chinese Academy of Medical Sciences, Beijing, China; ^2^ Key Laboratory of Bioactive Substances and Resources Utilization of Chinese Herbal Medicine, Ministry of Education, Beijing, China; ^3^ Key Laboratory of New Drug Discovery Based on Classic Chinese Medicine Prescription, Chinese Academy of Medical Sciences, Beijing, China; ^4^ China Pharmaceutical University, Jiangsu, China; ^5^ Beijing University of Chemical Technology, Beijing, China

**Keywords:** ischemic stroke, notoginsenoside R1, neurogenesis, neurological recovery, oligodendrogenesis

## Abstract

Notoginsenoside R1 (R1), a major component isolated from *P. notoginseng*, is a phytoestrogen that exerts many neuroprotective effects in a rat model of ischemic stroke. However, its long-term effects on neurogenesis and neurological restoration after ischemic stroke have not been investigated. The aim of this study was to evaluate the effects of R1 on neurogenesis and long-term functional recovery after ischemic stroke. We used male Sprague-Dawley rats subjected to middle cerebral artery occlusion/reperfusion (MCAO/R). R1 was administered by intraperitoneal (i.p.) injection immediately postischemia. We showed that R1 significantly decreased infarct volume and neuronal loss, restored neurological function, and stimulated neurogenesis and oligodendrogenesis in rats subjected to MCAO/R. More importantly, R1 promoted neuronal proliferation in PC12 cells *in vitro*. The proneurogenic effects of R1 were associated with the activation of Akt/cAMP responsive element-binding protein, as shown by the R1-induced increase in brain-derived neurotrophic factor (BDNF) expression, and with the activation of neurological function, which was partially eliminated by selective inhibitors of BDNF and PI3K. We demonstrated that R1 is a promising compound that exerts neuroprotective and proneurogenic effects, possibly via the activation of BDNF/Akt/CREB signaling. These findings offer insight into exploring new mechanisms in long-term functional recovery after R1 treatment of ischemic stroke.

## Introduction

Stroke is the second most common cause of human death and the leading cause of human disability with high morbidity worldwide (O'Donnell et al., 2010; Abdulhak et al., 2014). Due to the narrow window for administering rtPA (recombinant tissue plasminogen activator) treatment, only a small percentage of patients receive rtPA treatment during this therapeutic window (4.5 h) after the onset of stroke (Dibajnia and Morshead, 2013; Dirnagl and Endres, 2014). Therefore, new drugs that target the subacute and chronic stages of stroke are urgently needed. In the ischemic area, neurons die quickly due to a series of biochemical changes. Therefore, enhancing the survival of newborn neurons is an attractive strategy for promoting neurogenesis after ischemic stroke (Wang et al., 2016b).

Ischemic stroke induces substantial neurogenesis in the two specific regions of the adult brain: the subventricular zone (SVZ), which lines the lateral ventricular wall (LV), and the subgranular zone (SGZ) of the dentate gyrus (DG) (Kempermann and Gage, 1999; Ming and Song, 2011); these regions are the sites where most of the neural progenitors in the adult mammalian brain are located (Gross, 2000). Neuronal stem cells located in the SVZ mainly produce committed progenitor cells that migrate into the olfactory bulb (OB) through the rostral migratory stream and differentiate into local interneurons (Sharma and Reed, 2013); progenitor cells located in the SGZ migrate mainly to the granular cell layer and differentiate into neurons (Eriksson et al., 1998). After ischemic injury, the migration of newborn neurons is not confined to these constant sites (Jin et al., 2004). In response to ischemic injury, endogenous neuron precursors gradually migrate to the striatum, the hippocampus CA1 region and ischemic core area of the cerebral cortex, where they can merge with brain circuits and complete neural repair processes (Arvidsson et al., 2002; Jin et al., 2003). Newborn neurons have crucial physiological functions in learning and memory, plasticity and mood regulation. Therefore, a drug that effectively promotes the survival and formation of newborn neurons would provide a novel therapeutic strategy for ischemia-induced neurological damage; the search for such a drug remains the focus of both basic and clinical research.

*Panax notoginseng* is a famous traditional Chinese herb that has great clinical value for regulating cardiovascular (Lei and Chiou, 1986) and cerebrovascular (Su et al., 2014) diseases in China. Notoginsenoside R1 (R1), a major component isolated from *P. notoginseng*, is a novel phytoestrogen that exerts many neuroprotective effects in a rat model of ischemic stroke through the suppression of oxidative stress (Meng et al., 2014), apoptosis (Zou et al., 2017) and endoplasmic reticulum stress (ERS) (Wang et al., 2016a). Our previous studies mainly revealed the neuroprotective effects of pretreatment with R1 at the acute stage of stroke in rats (Meng et al., 2014), and its long-term effects on neuronal regeneration and neurological restoration after ischemic stroke have not been investigated.

In the present study, we used *in vivo* and *in vitro* models of cerebral ischemic/reperfusion (I/R) injury for MCAO/R in rats and oxygen-glucose deprivation/reoxygenation (OGD/R) in PC12 cells. The primary purpose of the present study was to evaluate the effects of R1 on neurogenesis and long-term functional recovery after ischemic stroke. Moreover, the mechanisms by which R1 facilitated neurogenesis in rats subjected to MCAO/R we were elucidated.

## Materials and Methods

### Materials

R1 (molecular weight = 933.15; purity >98%) was purchased from Shanghai Winherb Medical S and T Development (Beijing, China). A positive drug dl-3-n-butylphthalide (NBP) was obtained from CSPC NBP Pharmaceutical Co., Ltd. Edaravone was provided by Kunmingshenghuo Pharmaceutical Co., Ltd. Triphenyltetrazolium chloride (TTC) and 1,5-DAN hydrochloride were purchased from Sigma-Aldrich (MO, United States). 5-Ethynyl-2′-deoxyuridine (EdU) was purchased from Invitrogen (Grand Island). An EdU cell proliferation kit was purchased from Beyotime Biotechnology (Shanghai, China). All the primary antibodies used in the experiments were provided by Abcam (Cambridge, UK). The ANA-12 and LY294002 inhibitors and the ELISA kits for BDNF, nerve growth factor (NGF), and neurotrophin-4 (NT-4) were acquired from HaiTai TongDa Sci Tech Ltd (Beijing, China). Dulbecco’s modified Eagle’s medium (DMEM) and fetal bovine serum (FBS) were obtained from Gibco (Grand Island, NY, United States).

### Animals

Male Sprague-Dawley (SD) rats (purchased from Beijing Vital River Laboratories, Beijing, China) weighing 250–280 g were used in this study. All rats care and experimental procedures were reported in accordance with the Laboratory Animal Ethics Committee of the Institute of Medicinal Plant Development, Peking Union Medical College and complied with NIH Guidelines for the Care and Use of Laboratory Animals (approval number: SYXK 2017–0020). All rats were maintained in ventilated cages at a temperature of 20–25°C and a relative humidity of 30–50% under a 12 h light/dark cycle and were given free access to food and water.

### MCAO Surgery

The SD rats were anesthetized with ketamine (80 mg kg^−1^) and xylazine (10 mg kg^−1^) intraperitoneally (i.p.) by using the MCAO procedure. Cerebral I/R was induced by MCAO/R as previously described (Meng et al., 2014). After MCAO surgery, the wound was disinfected with iodine, and then the wound was sutured with sterile surgical suture to reduce the bleeding. We also injected tramadol (2.5 mg kg^−1^) by tail intravenous to relieve the pain caused by the operation. The sham-operated rats were manipulated using the same surgical procedure, but the MCA was not occluded. The body temperature was maintained at 37 ± 0.5°C until rats woke up using a heating pad (sunbeam, United States). The researcher who conducted all the subsequent analyses was blinded to the treatment that the rats had received.

### Drug Treatment

**FIGURE 1:**
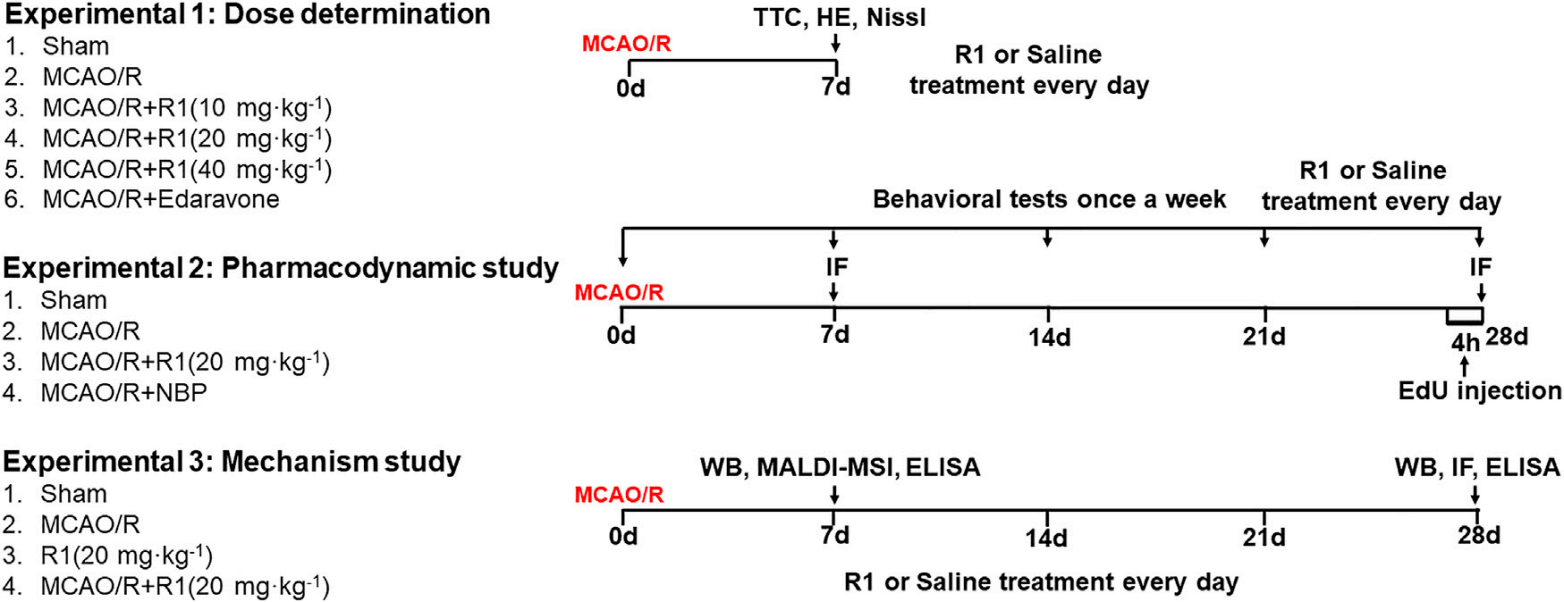
Schematic graphic of the rat experimental design. On the left is the group information, and on the right is the timeline of the experimental process.

The drug was dissolved in 0.9% normal saline prior to administration. The drug was administered by i. p. injection. To select the drug dosages, R1 was given at doses of 10 mg kg^−1^, 20 mg kg^−1^ and 40 mg kg^−1^ for 7 days after MCAO surgery. To detect neurogenesis, the optimal drug dosage was given for 28 days after MCAO surgery. At 28 days post-injection, serum and brain tissues were harvested to investigate the mechanism underlying the R1-mediated regulation of ischemic stroke in rats subjected to MCAO/R. Schematic graphic of drug treatment refers to Figure 1.

### Neurological Score

Neurological behavior was investigated at 1, 7, 14, 21, and 28 days after I/R by two blinded investigators using a 5-point scale as previously published (Meng et al., 2014). The neurological function was scored according to a series of scales from 0 to 4. The highest score represents the most severe neurological deficits.

### TTC Staining

TTC staining was conducted 7 days after I/R based on previously described methods (*n* = 5 for each group) (Meng et al., 2014). Cerebral infarct area was quantified by an image analysis system (Image-Pro Plus 5.0). The infarct volume can be obtained by multiplying the total infarct area by the thickness of the brain sections. Calculating the corrected infarct volume is contribute to compensate for the error caused by brain edema (Meng et al., 2014).

### Novel Object Recognition Task

The novel object recognition test (NORT) was performed as previously described (Camarasa et al., 2010; Zhang et al., 2018). To assess nonspatial memory, the rats were familiarized with an open-field box (50 × 50 × 50 cm, length × width × height) 28 days after MCAO/R to reduce the contribution of stress and anxiety. In the first phase, a learning trial was conducted for 10 min. In this phase, two objects of the same shape, color and size were placed symmetrically in the open field approximately 6 cm from the walls. In the second phase, one of the objects was replaced with a novel object of a dissimilar shape and color. During the 10 min recall trial, these two objects were presented in the same box 1 h after the first trial. All the objects and boxes were wiped down with 75% ethanol after each individual trial to avoid olfactory cues. Notebook computers recorded the time spent exploring the same object (TA1) and the novel object (TB1). The discrimination index was evaluated according to the following expression (TA1-TB1/TA1+TB1).

### Cylinder Tests

The cylinder tests were performed as previously described (Huotarinen et al., 2018). To assess asymmetric forelimb use during spontaneous vertical movements, the rats were tested individually in a 25 cm diameter glass cylinder, and videos were recorded for 5 min. The number of times the paw contralateral to the lesion contacted the supporting wall was counted and was expressed as a percentage of all the supporting wall contacts in each session (Francardo et al., 2014).

### Histopathology Staining

Histopathological staining (7 days postreperfusion) was conducted based on previously described methods (Wang et al., 2016c). The brain samples were embedded in paraffin and coronally dissected into 5 μm-thick sections. Then paraffin sections were stained with H&E and Nissl staining to reveal the histopathological lesions.

### EdU Injection and Immunofluorescence Staining

The relative numbers of proliferating cells were identified by double immunofluorescence staining with the relevant primary antibodies and EdU, which is a marker of cell proliferation. At 1 and 4 weeks after reperfusion, some rats received a single intraperitoneal injection of EdU (100 mg kg^−1^). After 4 h, these rats were transcardially perfused with PBS, and the tissue was fixed in 4% paraformaldehyde solution overnight (*n* = 3 per group). For double immunofluorescence staining, the frozen sections were incubated for 15 min in 0.3% Triton X-100 to disrupt the cell membrane, and then, the sections were incubated with anti-neuron-specific nuclear protein (NeuN, 1:500; Abcam, Cambridge, UK), anti-Doublecortin (DCX, 1:500; Abcam, Cambridge, UK), anti-Nestin (1:500; Abcam, Cambridge, UK) and anti-adenomatous polyposis *coli* (APC, 1:500; Abcam, Cambridge, UK) in blocking serum overnight at 4°C. After washing, the sections were incubated with FITC-conjugated secondary antibodies for 1 h and sealed with a coverslip. Then, the sections were incubated with a BeyoClick™ EdU-594 reaction cocktail for 30 min for EdU staining. Images were observed using TissueFAXS (Zeiss, Germany). Immunofluorescent positive cells were counted in three sections per rat. Results were expressed as the average numbers of positive cells in unit area per section of three rat brains.

### Mass Spectrometry Imaging

Frozen sections (10 μm) of the brain tissues (*n* = 5 per group) were taken for MALDI-MSI using a Leica CM1950 cryostat (Leica Microsystems GmbH, Wetzlar, Germany) −20°C and placed the thawed brain tissue slices on electrically conductive glass slides coated with indium tin oxide (ITO). The MALDI MSI experiments were implemented using the Autoflex Speed™ MALDI-TOF/TOF MS (Bruker Daltonics, Bremen, Germany) as described reported previously (Liu et al., 2017).

### Cell Culture and Drug Preparation

PC12 cells were differentiated into neural cells by incubation with NGF (50 ng/ml; New England Biolabs, MA, United States) and were cultured in DMEM supplemented with 10% horse serum and 5% FBS at 37°C in an incubator. In all the experiments, PC12 cells in the exponential phase were used. The R1 stock solution (100 mM) was prepared with DMSO. The indicated concentrations of R1 were prepared immediately before use.

### Oxygen-Glucose Deprivation/Reoxygenation and Drug Treatment

OGD/R was conducted in PC12 cells to mimic cerebral I/R injury *in vitro*. This procedure was conducted according to a previously described method with slight modification (Lu et al., 2011; Meng et al., 2014). Briefly, the PC12 cells were cultured in glucose-free Locke’s medium under hypoxic conditions for 4 h. Then, the cells were moved from the anaerobic chamber (TYPE c; coy Laboratory Products, Inc, Grasslake, MI, United States) to a normoxic environment, the medium was replaced with normal medium, and the cells were allowed to reoxygenate for 12 h. In the R1-treated group, the PC12 cells subjected to ODG/R were treated with R1 (12.5–100 μM) for 12 h. In the inhibitor-treated group, the cells were preincubated with 10 µM ANA-12 and 10 µM LY294002 for 0.5 h prior to treatment with R1.

### Cell EdU Staining

Cell proliferation was examined with an EdU cell proliferation kit according to the recommended procedure. The photographs were acquired with a fluorescence microscope (Leica DM4000, Frankfurt, Germany).

### Enzyme-Linked Immunosorbent Assay

Enzyme-linked immunosorbent assay (ELISA) kits were used according to the manufacturer’s instructions to quantify the expression of BDNF (HT100026), NGF (HT100169), and NT-4 (HT100171) in the serum and brain tissue.

### Western Blot Analysis

Western blot analysis was conducted as previously described (Meng et al., 2014). Right cortex tissues were collected from each rat (*n* = 3), and the total protein was extracted using a protein extraction reagent supplemented with protease and phosphatase inhibitor cocktails (ComWin Biotech, China). The total protein concentration of each sample was determined by a BCA kit (ComWin Biotech, China). Equal amounts of protein were separated using SDS-polyacrylamide gel electrophoresis and were transferred to nitrocellulose membranes. These membranes were blocked before being incubated overnight at 4°C with the appropriate primary antibodies: cnpase (ab183500, 1:1000), MBP (ab209328, 1:1000), Vimentin (ab92547, 1:10,000), SYP (ab32127, 1:1000), PSD95 (ab76115, 1:1000), MAP-2 (ab32454, 1:2000), Tau-1 (ab75714, 1:1000), BDNF (ab108319, 1:1000), *p*-TrkB (Tyr705) (ab229908, 1:1000), TrkB (CST4603, 1:1000), *p*-CREB (Ser133) (ab32096, 1:1000), CREB (ab32515, 1:1000), *p*-Akt (Ser473) (CST4060, 1:1000), Akt (CST4685, 1:1000), and *β*-actin (EXP0036 F, 1:2000). Then, the membranes were washed three times and incubated with the appropriate secondary antibodies. The blots were visualized using enhanced chemiluminescence western blot detection kits (ComWin Biotech, China). The blot densities were calculated by ImageJ software.

### Statistical Analysis

Experimental data were obtained from three independent experiments and are expressed as the means ± standard deviations (SDs). All analyses were statistically evaluated using SPSS17 software (IBM Corporation, New York, NY, United States). One-way ANOVA followed by Tukey test or two-way ANOVA followed by Bonferroni’s multiple comparison test, if these data were normally distributed. The Kruskal-Wallis test was used if these data were not normal distributed. A *p* value of less than 0.05 was considered statistically significant.

## Results

### R1 Attenuates the Infarction Volumes and Neuronal Loss After Ischemia

**FIGURE 2:**
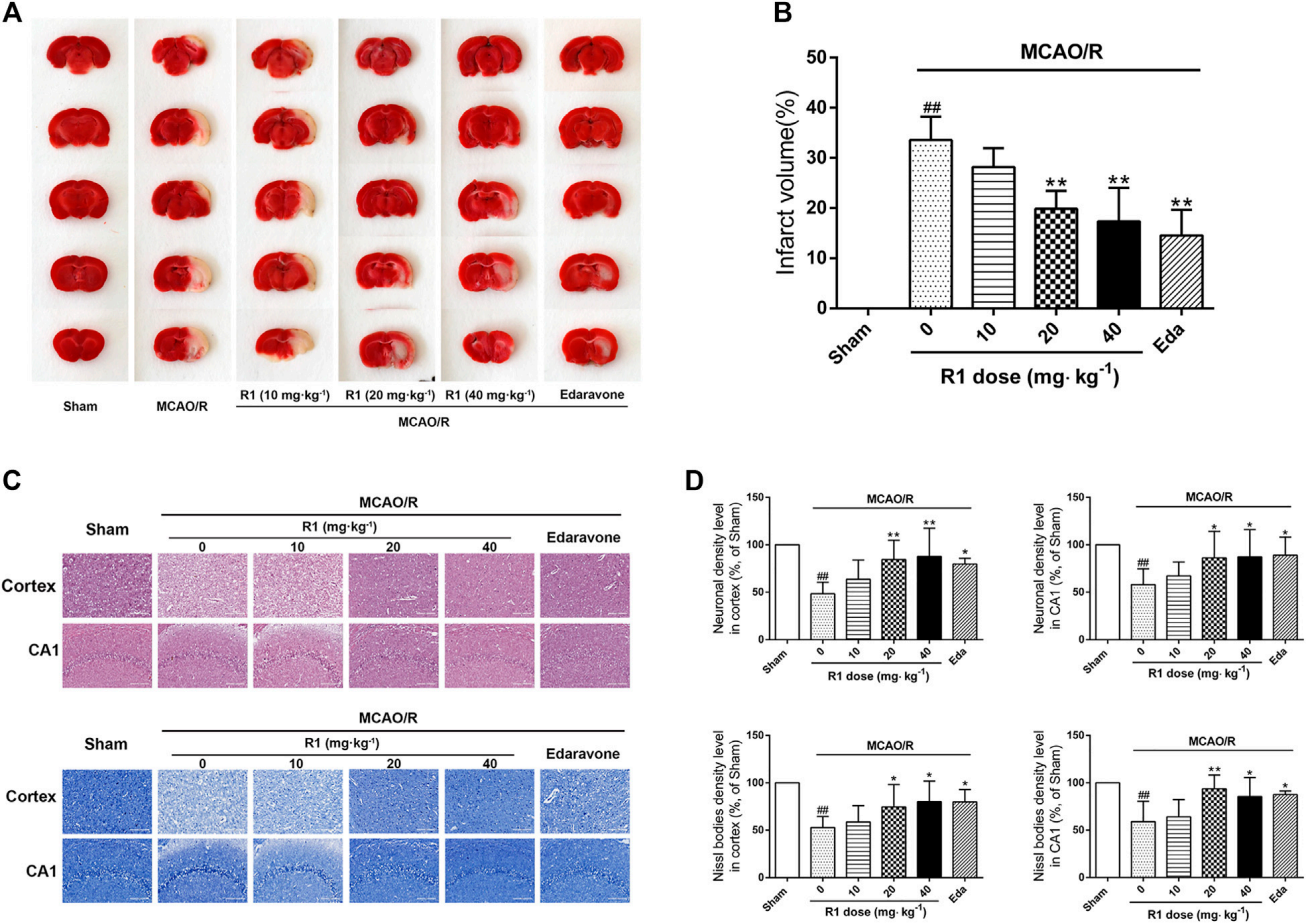
R1 reduces the infarction volume and neuronal loss in rats subjected to MCAO. R1 and edaravone were both intraperitoneally injected immediately after MCAO surgery **(A)** Effects of R1 on infarction volumes (n = 5) **(B)** Quantitative analysis of cerebral infarct volumes **(C)** H&E staining and Nissl staining of the cortex and hippocampal CA1 regions of each group (*n* = 5) **(D)** Relative density (% of sham) of the H&E and Nissl staining in the cortex and hippocampal CA1 regions in all the groups. Scale bar = 200 μm. Data are expressed as the mean ± SD and were analyzed by ANOVA. ^#^ *p*< 0.05 and ^##^ *p*< 0.01 vs. Sham group; *p< 0.05 and **p< 0.01 vs. MCAO/R group.

MCAO was performed on the right side. 7 days after ischemia, the infarction volumes and neuronal numbers were altered. After treatment for 7 days, 20 and 40 mg kg^−1^ R1 significantly reduced the infarction volumes and neuronal loss (Figures 2A–D). At a dose of 20 mg kg^−1^, R1 exhibited more remarkable effects of Nissl body loss on the hippocampal CA1 region. Thus, subsequent studies in the rats were performed with this dose of R1.

### R1 Ameliorates the Long-Term Neurological Dysfunction After Ischemia

**FIGURE 3:**
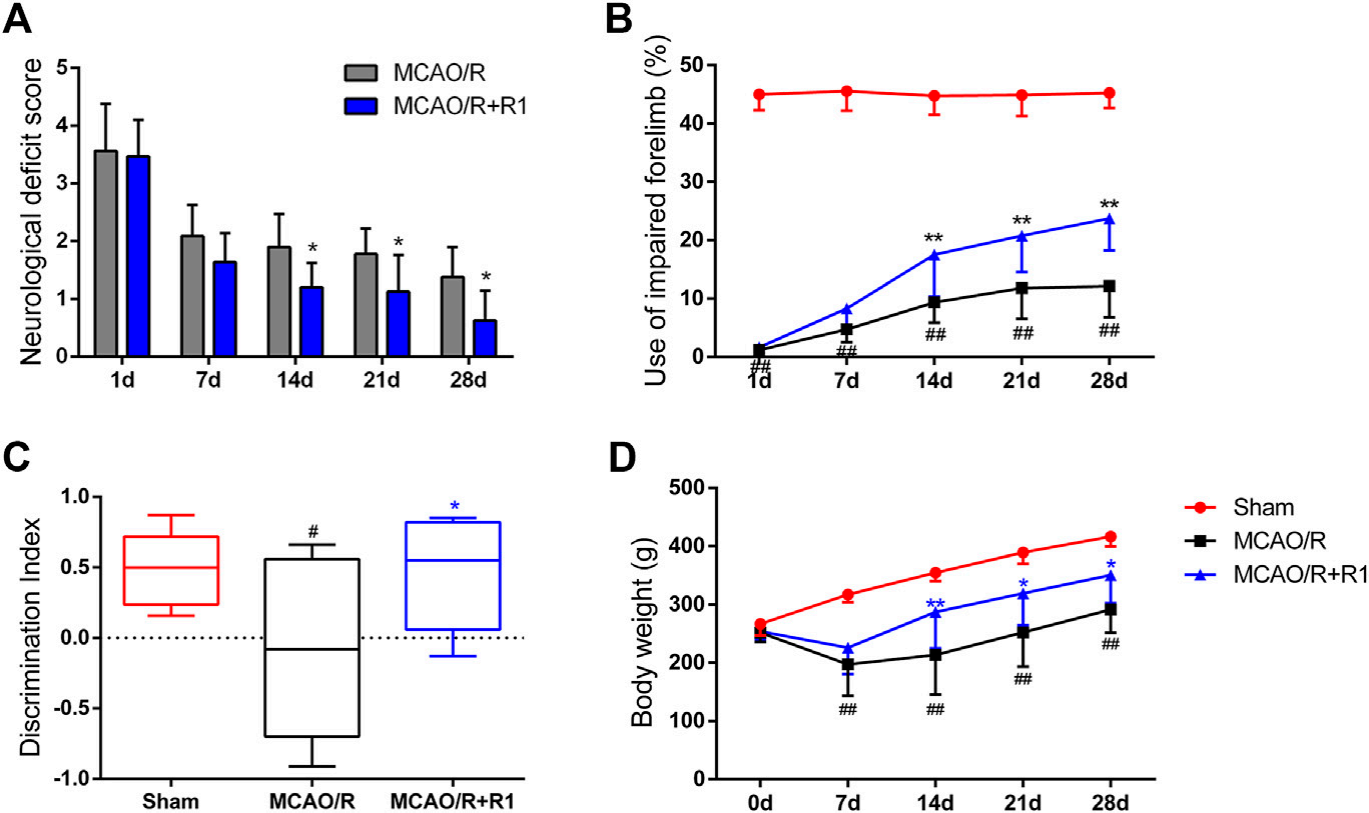
R1 restores long-term neurological function in rats subjected to MCAO. The behavioral tests were conducted on days 1, 7, 14, 21 and 28 after MCAO surgery **(A)** Zea-Longa score **(B)** cylinder tests **(C)** novel object recognition tests on day 28 after MCAO surgery **(D)** The body weight of the rats in each treatment group over 28 days. Data are expressed as the mean ± SD and were analyzed by ANOVA. ^#^ *p*< 0.05 and ^##^ *p*< 0.01 vs. Sham group; *p< 0.05 and **p< 0.01 vs. MCAO/R group.

To further evaluate the effect of R1 treatment on the long-term recovery of neurological function, a series of neurological assessments, including the Zea-Longa score, cylinder tests and novel object recognition tests, were performed throughout the 28 days observation period. All the rats subjected to MCAO presented consistent, substantial neurological deficits 1 day after MCAO surgery, and neurological function gradually improved during the 28 days. The neurological deficit scores of the rats in the R1 treatment group were significantly lower than those of the rats in the MCAO/R group 14 days after treatment (Figure 3A). The rats treated with R1 also exhibited significantly improved function of the impaired forelimb beginning at 14 days compared with the rats subjected to MCAO/R (Figure 3B). Moreover, in the NORT, R1 significantly increased the abilities of the rats to distinguish novel from familiar objects, as determined by the discrimination index (Figure 3C), indicating that R1 has the potential to improve recognition memory. Concomitantly, compared with the MCAO/R group, the R1-treated group exhibited an increase in body weight (Figure 3D).

### R1 Promotes Regenerative Neurogenesis After Ischemia

**FIGURE 4:**
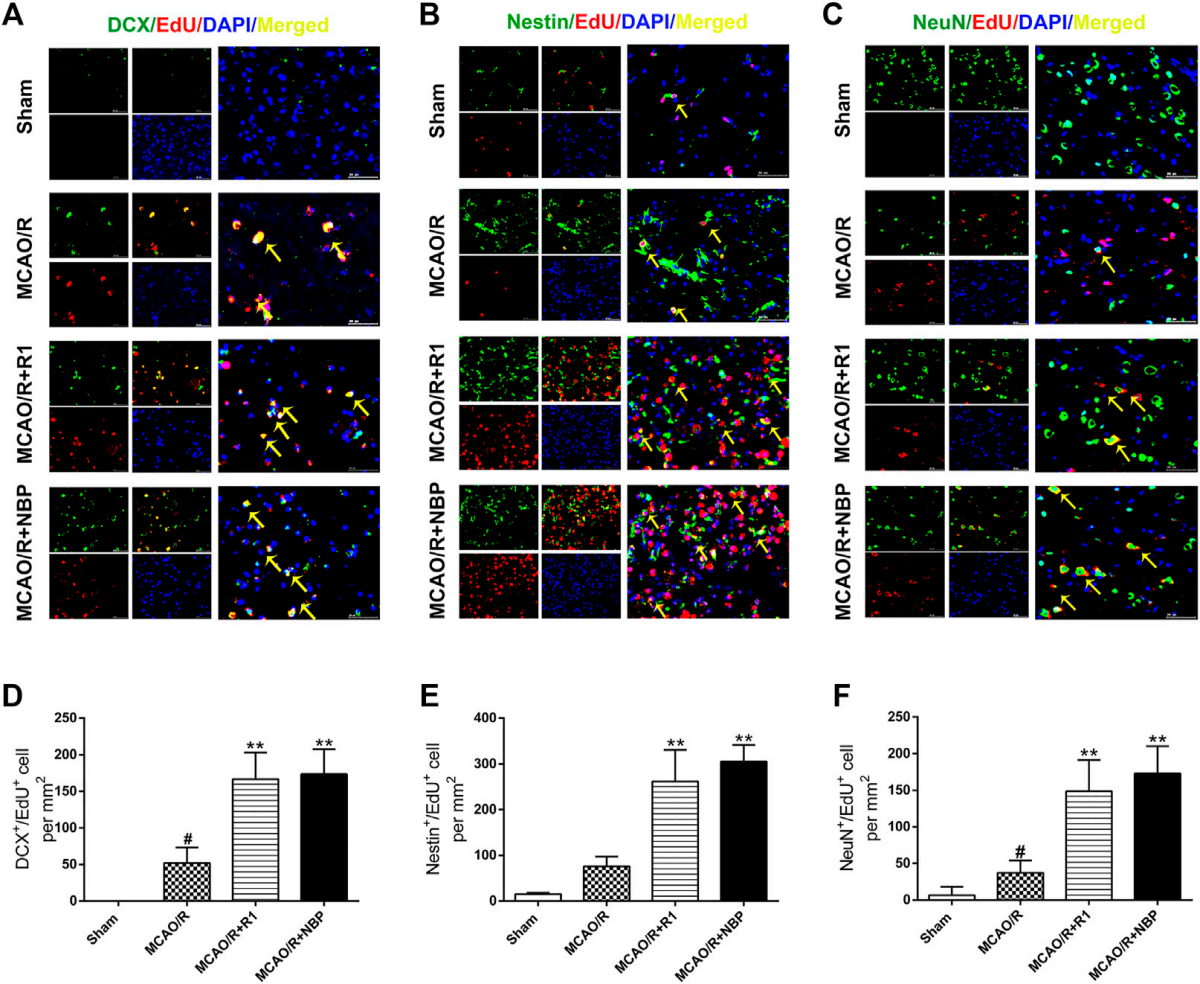
R1 enhances neural reconstruction by stimulating neurogenesis after ischemic stroke. Representative images of the infarction area costained with antibodies against **(A)** DCX (green, marker of migrating and immature neurons) and EdU (red, marker of proliferating cells) on day 7 after R1 treatment, yellow arrows indicate migrating neuroblasts (EdU^+^/DCX^+^ cells) **(B)** Nestin (green, marker of proliferating NPCs) and EdU (red) on day 7 after R1 treatment, yellow arrows indicate proliferating NPCs (EdU^+^/Nestin^+^ cells) **(C)** NeuN (green, marker of mature neurons) and EdU (red) on day 28 after R1 treatment, yellow arrows indicate newly formed mature neurons (EdU^+^/NeuN^+^ cells). DAPI (blue) indicates the nucleus, Scale bar = 50 μm **(D-F)** Quantitative analysis of **(A-C)** in the striatum region respectively after R1 treatment. *n* = 3 brains per group. Data are expressed as the mean ± SD and were analyzed by ANOVA. ^#^ *p*< 0.05, ^##^ *p*< 0.01 vs. Sham group; ^*^ *p*< 0.05, **p< 0.01 vs. MCAO/R group.

After MCAO surgery, the neuron structures and numbers were destroyed. To confirm whether R1 possesses long-term therapeutic effects postischemia on the migration and proliferation of newborn neurons, we performed DCX/EdU, Nestin/EdU and NeuN/EdU double staining to identify neuroblasts (migrating and immature neurons) (Gleeson et al., 1999), proliferating NPCs (Arvidsson et al., 2002) and newborn mature neurons, respectively. The striatum and cortex regions in the brain are commonly considered to be sensitive to cerebral ischemia (Zhang et al., 2018). In the sham group of our study, almost no DCX^+^/EdU^+^, Nestin^+^/EdU^+^ or NeuN^+^/EdU^+^ cells were detected in the striatum region. However, in the rats subjected to MCAO, the numbers of DCX^+^/EdU^+^, Nestin^+^/EdU^+^ and NeuN^+^/EdU^+^ cells were significantly increased in the infarcted area of the ipsilateral hemisphere. Furthermore, the numbers of DCX^+^/EdU^+^ (Figures 4A,D), Nestin^+^/EdU^+^ (Figures 4B,E) and NeuN^+^/EdU^+^ (Figures 4C,F) double-positive cells were obviously increased after R1 treatment compared with MCAO treatment.

### R1 Stimulates Oligodendrogenesis and Preserves Myelin After Ischemia

**FIGURE 5:**
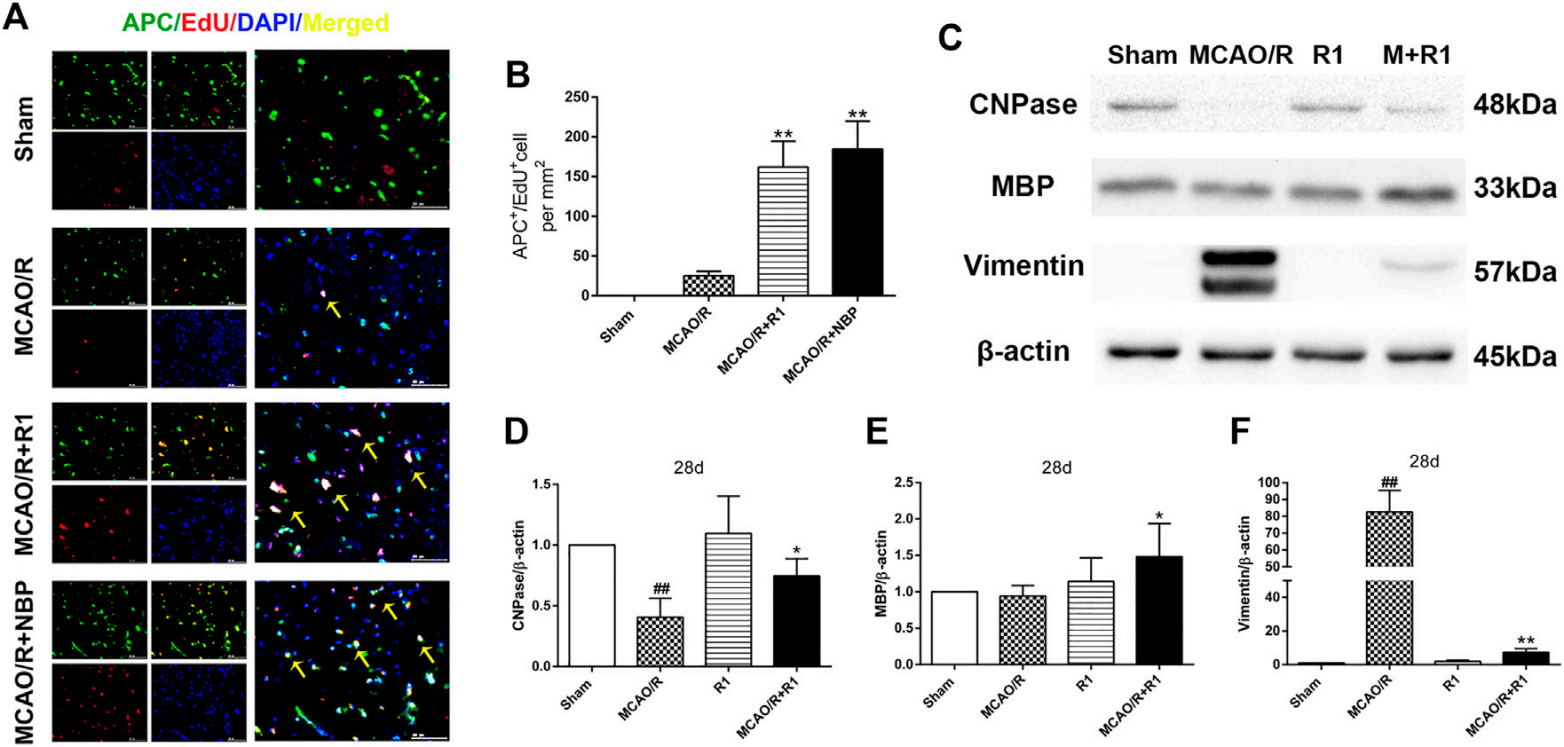
R1 facilitates oligodendrogenesis and preserves myelin after ischemic stroke **(A)** Representative images of the infarction area costained with antibodies against APC^+^ (green, marker of oligodendrocytes) and EdU (red, marker of proliferating cells) on day 28 after R1 treatment, yellow arrows indicate proliferated oligodendrocytes (EdU^+^/APC^+^ cells), DAPI (blue) indicates the nucleus, Scale bar = 50 μm **(B)** Quantitative analysis of proliferating oligodendrocytes (EdU^+^/APC^+^ cells) in the cortex region after R1 treatment. *n* = 3 brains per group. Representative images of immunoblotting **(C)** and quantification of cnpase (cyclicnucleotide 30-phosphohydrolase, **D**), MBP (myelin basic protein, **E**), and vimentin **(F)** in the infarct cortex region of the Sham, MCAO/R, R1, and MCAO/R + R1 groups on day 28 after MCAO surgery. n = 3 in each group. Data are expressed as the mean ± SD and were analyzed by ANOVA. ^#^ *p*< 0.05, ^##^ *p*< 0.01 vs. Sham group; ^*^ *p*< 0.05, **p< 0.01 vs. MCAO/R group.

Axon diameter and myelin thickness and spacing determine the rate of neuronal conduction along the axon. Oligodendrocyte precursor cells (OPCs) generate myelin-forming oligodendrocytes, which are essential for myelin regeneration and functional recovery after cerebral ischemia (Kang et al., 2010; Hughes et al., 2013; Hill et al., 2018). By double labeling with APC, which a marker of mature oligodendrocytes (Han et al., 2015), and EdU, we found that the group treated with R1 for 28 days exhibited significantly increased numbers of APC^+^/EdU^+^ cells in the infarcted area compared with the group subjected to MCAO/R (Figures 5A,B). Concomitantly, R1 treatment for 28 days elevated the protein expression of cnpase and MBP (Saneto and de Vellis, 1985), which are markers of immature oligodendrocytes. Moreover, the dramatic increase in the protein expression of the microglia and reactive astrocyte marker vimentin (Jiang et al., 2012) after ischemia was markedly reduced by R1 (Figures 5C–F).

### R1 Increases Neurotrophic Factor Expression and Promotes Synaptic Formation After Ischemia

**FIGURE 6:**
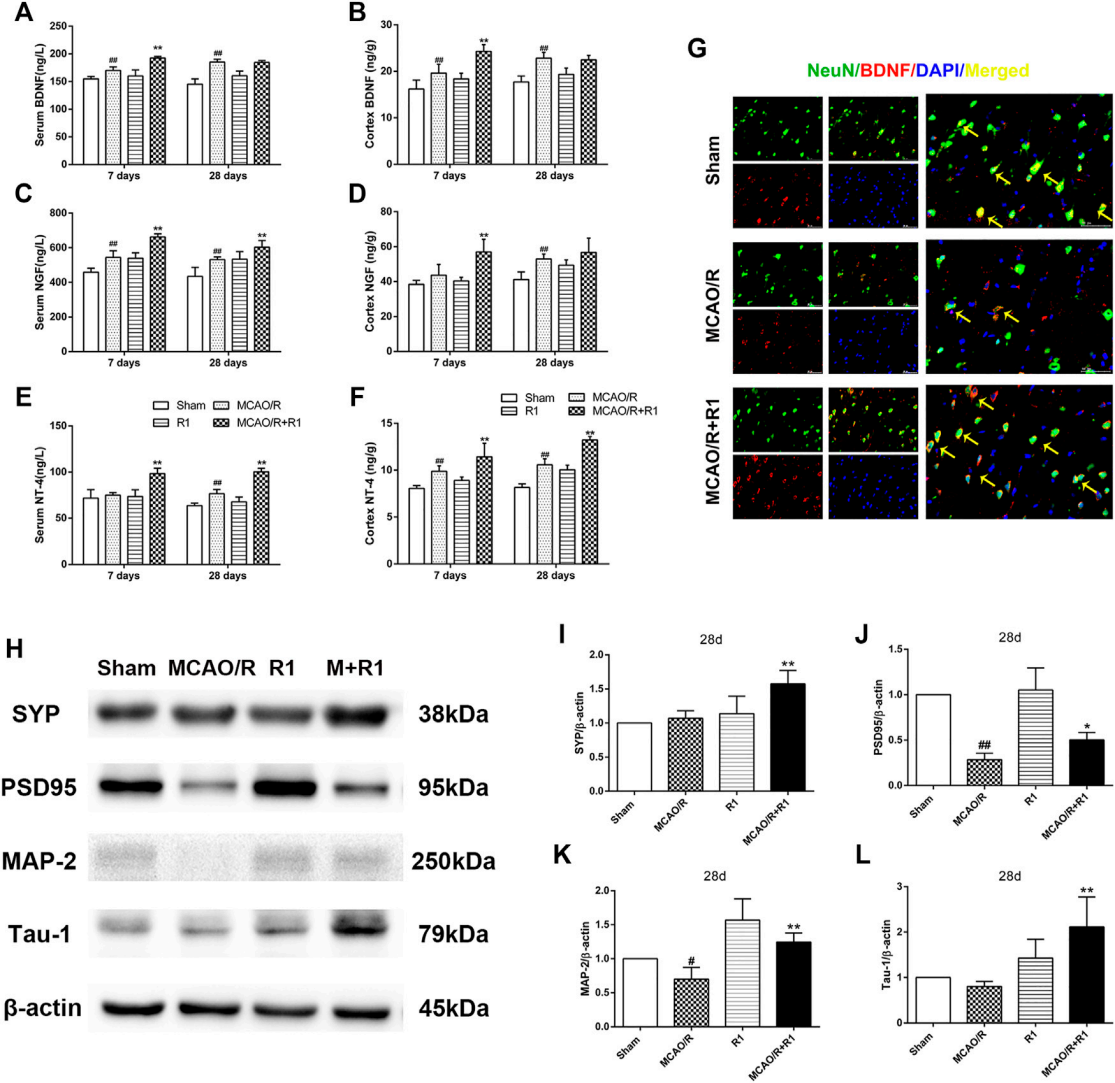
R1 increases neurotrophic factor expression and restores disrupted neural synaptic function after ischemic stroke. The serum and cortex tissue levels of neurotrophic factors, such as BDNF **(A, B)**, NGF **(C, D)**, and NT-4 **(E, F)** were detected by ELISA. **(G)** Representative images of the infarction area costained with antibodies against BDNF (red) and NeuN (green) on day 28 after R1 treatment, yellow arrows indicate NeuN-positive neurons expressed BDNF, DAPI (blue) indicates the nucleus, Scale bar = 50 μm. Representative images of immunoblotting **(H)** and quantification of the relative protein levels of SYN **(I)**, PSD95 **(J)**, MAP-1 **(K)** and Tau-1 **(L)** in the infarct cortex region of the Sham, MCAO/R, R1, and MCAO/R + R1 groups on day 28 after MCAO surgery. *n* = 3 in each group. Data are expressed as the mean ± SD and were analyzed by ANOVA. ^#^ *p*< 0.05, ^##^ *p*< 0.01 vs. Sham group; ^*^ *p*< 0.05, **p< 0.01 vs. MCAO/R group.

Neurotrophic factors play a major role in regulating neurite sprouting and regeneration in response to nerve injuries (Fornaro et al., 2020). By ELISA, we found that the group treated with R1 exhibited clearly increased protein levels of various neurotrophic factors, such as BDNF, NGF and NT-4, compared with the group subjected to MCAO (Figures 6A–F). BDNF is a neurotrophic factor known to regulate neuronal survival and growth and to actively participate in synaptic transmission and plasticity in various brain regions (Kang and Schuman, 1995; Ding et al., 2011). To determine how BDNF activity is affected by R1 after MCAO surgery, we located the main source of BDNF by double immunofluorescence staining. We reported that a few NeuN-positive neurons expressed BDNF 28 days after MCAO surgery. However, NeuN-positive neurons predominately expressed BDNF after R1 treatment for 28 days (Figure 6G). Moreover, the crucial question is whether R1 can promote synaptic formation after ischemic injuries. Here, we detected the protein expression of SYN (synaptophysin), PSD95 (postsynaptic density protein 95), MAP2 (a somato-dendritic marker) and Tau-1 (an axonal marker). We found that rats treated with R1 for 28 days exhibited dramatically higher expression of SYN, PSD95, MAP-2 and Tau-1 than those treated with saline (Figure 6H–L).

### R1 Promotes Regenerative Neurogenesis Through Akt/CREB Activation by Upregulating BDNF Expression

**FIGURE 7:**
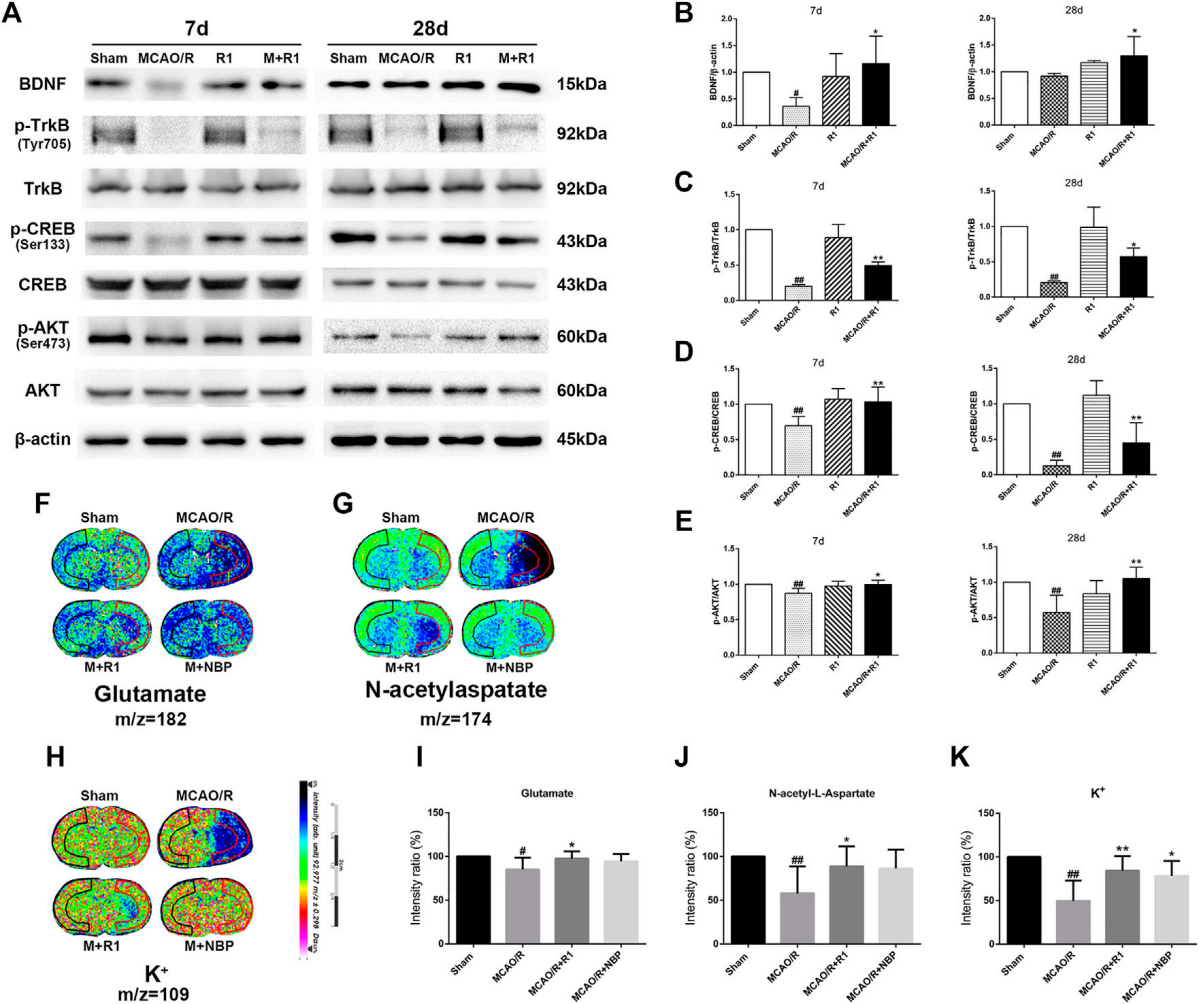
R1 activates the BDNF/Akt/CREB signaling pathway to promote the recovery of neurological function. Representative images of immunoblotting **(A)** and quantification of the relative protein levels of BDNF **(B)**, *p*-TrkB/TrkB **(C)**, *p*-CREB/CREB **(D)** and **(E)** *p*-Akt/Akt in the infarct cortex region of the Sham, MCAO/R, R1, and MCAO/R + R1 groups on days 7 and 28 after MCAO surgery. *n* = 3 in each group. *In situ* MALDI MSI of glutamate **(F)**, N-acetylaspatate **(G)** and K^+^ **(H) (I-K)** Quantitative measurement of the corresponding indicators in the ischemic cortex. Scale bar = 2 cm, *n* = 5 in each group. Data are expressed as the mean ± SD and were analyzed by ANOVA. ^#^ *p*< 0.05, ^##^ *p*< 0.01 vs. Sham group; ^*^ *p*< 0.05, **p< 0.01 vs. MCAO/R group.

Mature BDNF specifically activates its receptor, tyrosine receptor kinase B (TrkB), to promote the survival, growth, migration, differentiation and maturation of NPCs (Keifer et al., 2009; Song et al., 2015). We aimed to assess if BDNF and the downstream effectors of TrkB signaling are involved in the mechanisms underlying the R1-mediated promotion of neurogenesis. Western blotting assays of the ischemic cortical tissue revealed that the rats treated with R1 for 7 and 28 days exhibited dramatically increased BDNF expression compared with the rats subjected to MCAO (Figures 7A,B). Akt and CREB have been confirmed as downstream targets of BDNF (Clarkson et al., 2015). We also found that 7 and 28 days after ischemia, *p*-TrkB/TrkB, *p*-CREB/CREB and *p*-Akt/Akt were significantly decreased due to ischemic injury compared with the sham rats. In addition, at 7 and 28 days, the rats treated with R1 exhibited markedly increased *p*-TrkB/TrkB (Figures 7A,C), *p*-CREB/CREB (Figures 7A,D) and *p*-Akt/Akt (Figures 7A,E) expression compared with the rats subjected to MCAO.

The pathogenesis of neurological disorders, such as stroke, can disrupt the function or expression of neurotransmitters (Abg Abd Wahab et al., 2019; Vogt, 2019). Neurotransmitter release from synapses is affected by BDNF (Ding et al., 2011). We wondered if R1 can promote neurotransmitter release after ischemic injuries. By matrix-assisted laser desorption/ionization mass spectrometry imaging (MALDI–MSI), we found that glutamate, N-acetylaspatate and K^+^ profoundly were decreased 7 days after ischemic injury. The rats treated with R1 exhibited significantly higher levels of glutamate, N-acetylaspatate and K^+^ in the ischemic cortex region that the rats subjected to MCAO (Figures 7F–K), suggesting that R1 regulated synaptic communication by modulating neurotransmitters (Shariatgorji et al., 2019).

### R1 Promotes Neuronal Proliferation by BDNF and Akt Activation *in Vitro*

**FIGURE 8:**
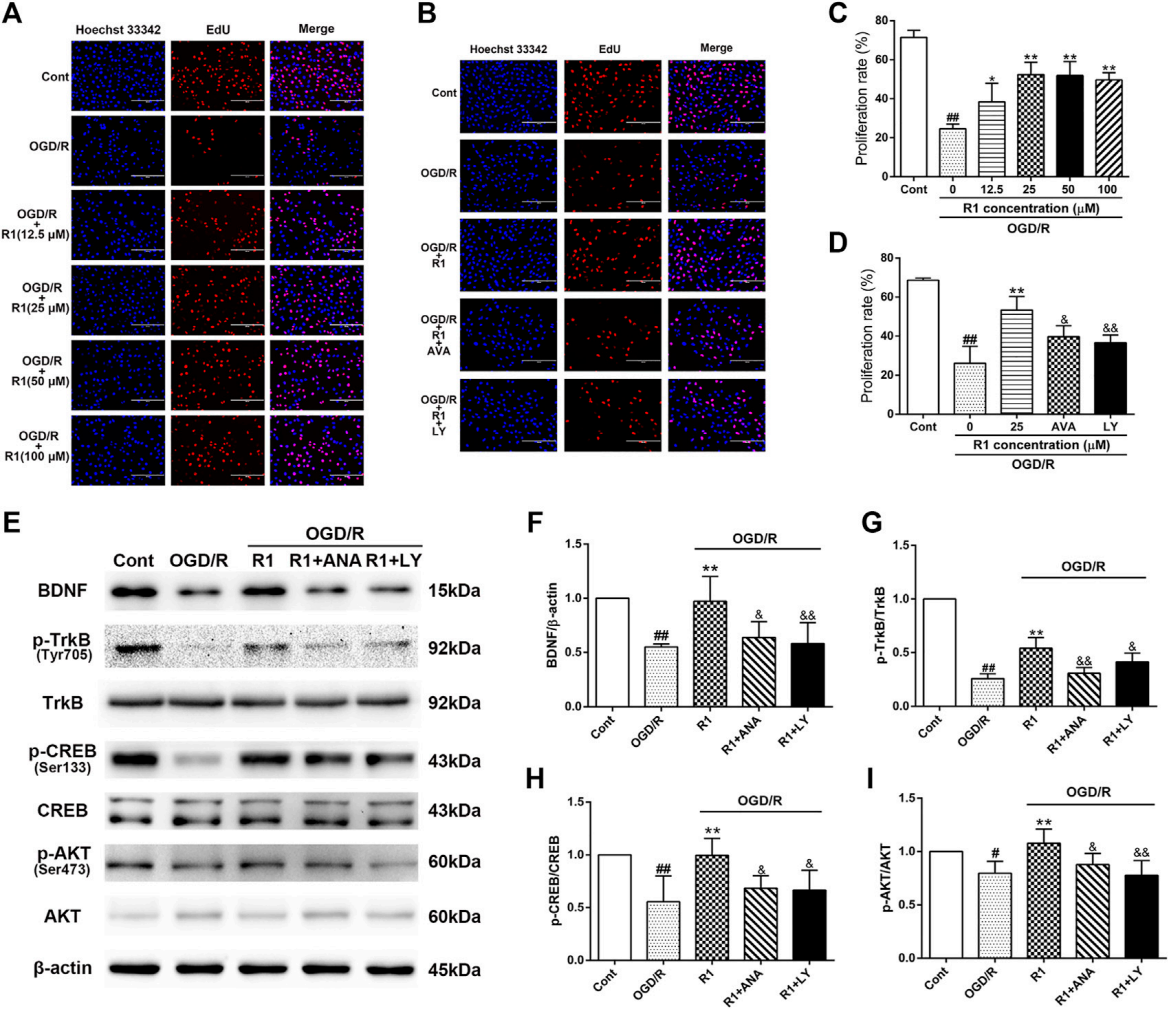
R1 promotes neuronal proliferation though the BDNF and PI3K signaling pathways *in vitro* **(A)** R1 promotes neuronal proliferation. PC-12 cells were treated with or without R1 (drug concentration reached up to 100 µM) for 12 h. Images of proliferating neurons detected by EdU staining. Nuclei were visualized by DAPI staining (blue). Scale bar = 200 μm **(B)** PC-12 cells were pretreated with or without R1 (25 µM) in the presence or absence of ANA-12 (10 μM) or LY294002 (10 μM) for 12 h. Images of proliferating neurons detected by EdU staining. Nuclei were visualized by DAPI staining (blue). Scale bar = 200 μm **(C, D)** Proliferation rate of EdU^+^ cells was analyzed. Representative images of immunoblotting **(E)** and quantification of the relative protein levels of BDNF **(F)**, *p*-TrkB/TrkB **(G)**, *p*-CREB/CREB **(H)** and *p*-Akt/Akt **(I)** in PC-12 cells. *n* = 3 in each group. Data are expressed as the mean ± SD and were analyzed by ANOVA. ^#^ *p*< 0.05, ^##^ *p*< 0.01 vs. Cont group; ^*^ *p*< 0.05, **p< 0.01 vs. OGD/R group; and *p*< 0.05, and *p*< 0.01 vs. R1 group.

To better determine the effect of R1 on neuronal proliferation and the potential mechanism involved, we incubated R1 (the concentration reached up to 100 µM) with PC12 cells (after the cell attachment rate was approximately 70%) for 12 h and examined neuronal proliferation by EdU staining. R1 (25, 50 and 100 μM) more notably promoted neuronal proliferation by increasing the proliferation rate of EdU^+^ cells (Figures 8A,C). To confirm whether the BDNF and Akt signals participated in the effect of R1 on neuronal proliferation, the neurons were pretreated with pharmacological inhibitors of the BDNF receptor TrkB (ANA-12) and PI3K (LY294002) before the addition of R1. Collectively, both ANA-12 and LY294002 inhibited the increased neuronal proliferation due to R1 (Figures 8B,D). Furthermore, both ANA-12 and LY294002 resulted in the loss of R1-induced neurogenesis after OGD/R injury and the R1-mediated preservation of BDNF, TrkB, CREB and Akt expression after OGD/R challenge, as shown by the western blotting results (Figures 8E–I).

## Discussion

Our previous studies have shown the neuroprotective effects of pretreatment with R1 during the acute stage of stroke in rats (Meng et al., 2014). In this study, we systematically investigated various doses of R1, injected i. p, and found that R1 decreased infarct volumes and ameliorated neurological deficits 7 days after ischemia in rats, suggesting that these doses produced optimal efficacies for evaluating neurological restoration after ischemic stroke.

We have demonstrated that R1 exerts potent neuroprotective effects via the suppression of NADPH oxidase- and mitochondrion-derived superoxide and the inhibition of oxidative stress level *in vivo* and *in vitro* (Meng et al., 2014). In this study, we focused on the long-term recovery of neurogenesis and oligodendrogenesis postischemic stroke in rats. R1 promoted the recovery of long-term neurological function and stimulated neurogenesis. These preliminary results provide a theoretical basis for the generation of newborn neurons in ischemic brains. To the best of our knowledge, newborn neurons play a key role in neural plasticity, learning and memory, and emotional regulation, and their dysregulation is involved in a variety of brain disorders (Christian et al., 2014; Anacker and Hen, 2017). Combined with our previous research (Meng et al., 2014), these results suggest that R1 has both neuroprotective and neurorestorative effects that lead to improved neurological function at both the acute and chronic phases postischemia.

Neuronal stem cells (neural progenitor cells, NPCs) present in the hippocampus SVZ and SGZ have the ability to self-renew and differentiate into neurons, astrocytes, and oligodendrocytes (Libert et al., 2008). After (hypoxic) ischemic brain injury, the rapid proliferation of young precursors enhances neurogenic capacity. In our present study, there was a significant increase in the number of DCX^+^/EdU^+^ cells and Nestin^+^/EdU^+^ cells in the striatum after R1 treatment, demonstrating that R1 stimulated the proliferation of NPCs and enhanced the formation and migration of neuroblasts. The vast majority of newborn neurons die within 2–5 weeks after ischemia due to the harmful niche environment, a lack of adequate trophic support, and the failure to form connections with other neurons, and only a small fraction of these newborn neurons can differentiate into mature neurons (Doeppner et al., 2011; Ryu et al., 2016). Our data also show that the number of NeuN^+^/EdU^+^ mature neurons in the periinfarct region was increased by R1 treatment. These results indicated that R1 treatment not only directly protects newborn neurons but also supports the long-term survival of newborn neurons by preserving the local microenvironment.

Axons and dendrites are the two main structural and functional units of differentiated mature neurons, and both synaptogenesis and dendritic remodeling are related to increases in neurological activity in the cerebral cortex (Dimyan and Cohen, 2011). Importantly, neurons that release neurotransmitters at the synapse can be considered the vocabulary of the neuronal language (Vogt, 2019). In our results, R1 upregulated the levels of various synapse and neurotransmitters, namely, PSD95, SYN, glutamate, N-acetylaspatate and K^+^, and increased the protein expression of mature dendrite and axon markers, namely, MAP-2 and Tau-1, respectively, which further showed that R1 is beneficial for promoting neuronal differentiation.

Oligodendrocytes originate from OPCs and eventually differentiate into myelin cells (Bergles and Richardson, 2015). The differentiation of oligodendrocytes and the subsequent myelination of axons can maintain axonal integrity and neuron survival, and the lack of this well-coordinated axon-glia interaction easily causes neuropsychiatric disorders (Herbert and Monk, 2017; Saab and Nave, 2017). Our work showed that the proliferation of APC^+^ cells after stroke was stimulated by R1 treatment. We also observed increased protein levels of cnpase, an immature oligodendrocyte-specific protein, and MBP, or myelin basic protein. These findings indicated that R1 has the ability to promote oligodendrocyte production and remyelination after I/R injury. These benefits provide therapeutic potential for treating multiple sclerosis, spinal cord injury and other demyelinating diseases (Najm et al., 2015; Saab and Nave, 2017).

Many neurotrophic factors, including BDNF, NGF, and NT-4 have been demonstrated to protect neural stem cells (NSCs) and to promote neurogenesis after cerebral ischemia (Edelbrock et al., 2018). Our current study revealed that R1 treatment significantly increased the expression of BDNF, NGF and NT-4 after ischemic stroke, and these results are similar to a previous report on the promotion of neurogenesis following the overexpression of adenoviral-transduced BDNF mRNA (Benraiss et al., 2001). Because the occurrence of ischemic stroke will increase the permeability of the blood brain barrier (BBB), a large number of inflammatory substances enter the brain to induce brain edema, accompanied by the production of newborn neurons. However, 80% of newborn neurons die due to lack of nutritional support or lack of timely drug intervention (Arvidsson et al., 2002). Our current study showed that R1 treatment remarkably alleviated BBB disruption (Supplementary Figure S1) and facilitated neurogenesis after I/R injury by increasing BDNF expression. Part of the reason was that the increased BDNF expression of R1 provides a microenvironment suitable for survival of newborn neurons. Simultaneously, the massive production of new neurons will repair the brain damage caused by ischemia, and then improve the permeability of the BBB.

BDNF specifically binds to TrkB receptor, which can promote neuron differentiation, maturation and synaptic plasticity during development or after injury (Edelbrock et al., 2018). These functions of BDNF/TrkB signaling are achieved by a combination of three downstream signaling cascades: the phosphatidylinositol 3-kinase (PI3K), mitogen-activated protein kinase (MAPK) and phospholipase-C gamma (PLCγ) pathways (Edelbrock et al., 2018). Moreover, the activation of BDNF/TrkB signaling results in the phosphorylation of the transcription factor CREB (Shang et al., 2019; Du et al., 2020). Blocking BDNF signaling leads to reduced CREB transcription, which is essential for synaptic plasticity and learning and memory (Mowla et al., 1999). Western blot analysis confirmed that R1 upregulates the protein expression of BDNF, *p*-TrkB, *p*-Akt and *p*-CREB and that ANA-12 (TrkB inhibitor) and LY-294002 (PI3K inhibitor) significantly inhibited the R1-induced neural migration and proliferation *in vitro*. The data suggest that R1 promotes the recovery of neurological function after stroke via the BDNF/Akt/CREB signaling pathway. A previous study showed that Akt phosphorylates CREB, resulting in CREB-mediated gene expression, including the expression of BDNF, that is essential for neuron survival (Lu et al., 2013; Zhang et al., 2018). More specifically, we confirmed that BDNF, Akt and CREB participate in one regulatory loop. These findings were supported by the inhibition of TrkB (by ANA-12) and PI3K (by LY-294002), which resulted in the impairment of the R1-induced *p*-Akt and BDNF protein expression that ameliorated the I/R-induced neurofunction deficits.

## Conclusion

**FIGURE 9:**
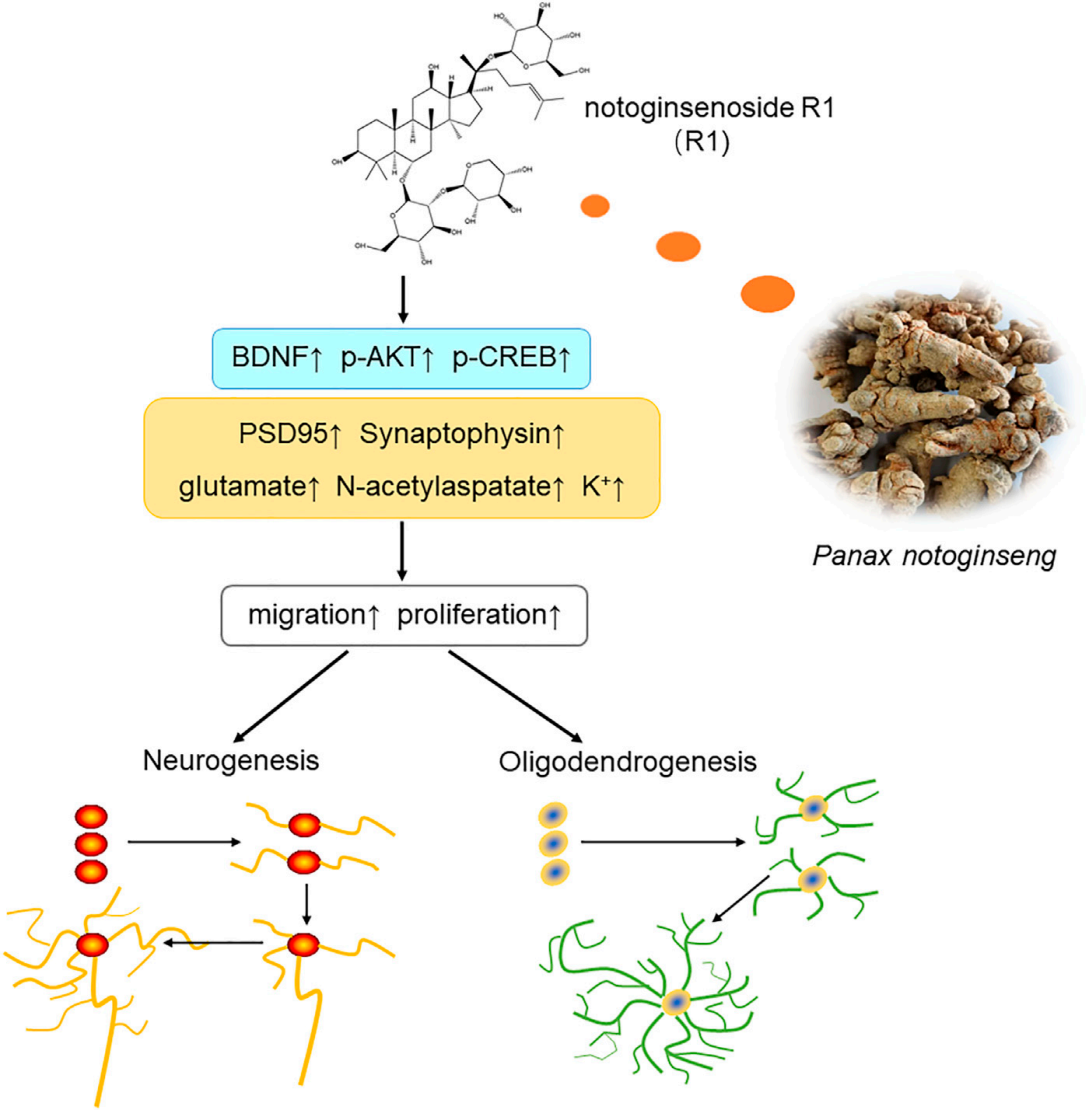
Schematic diagram of the mechanisms underlying the R1-induced promotion of neurological recovery after ischemia-reperfusion injury. Overall, R1 enhances neurogenesis and oligodendrogenesis by promoting NPC proliferation, migration and the survival of newborn neurons. Therefore, R1 stimulates the expression of neural synaptic factors and facilitates the release of neurotransmitters. R1 activates the BDNF/Akt/CREB signaling pathway, thereby accelerating neurogenesis to promote neurological recovery.

In summary, the current study showed that treatment with R1 promoted neurogenesis and oligodendrogenesis after ischemic stroke. The mechanisms by which R1 restored neurological function involved the upregulation of Akt/CREB by increasing the expression of BDNF (Figure 9). These findings demonstrated that R1 is a promising new treatment for the long-term recovery of neurological function after ischemic stroke by promoting neurogenesis and oligodendrogenesis.

## Data Availability

The original contributions presented in the study are included in the article/Supplementary Material, further inquiries can be directed to the corresponding authors.
