# Supplementary material for: Notoginsenoside R1 Improves Cerebral Ischemia/Reperfusion Injury by Promoting Neurogenesis via the BDNF/Akt/CREB Pathway
Source: Front Pharmacol. 2021 May 7;12:615998. doi: 10.3389/fphar.2021.615998 (PMC8138209; doi:10.3389/fphar.2021.615998)
Supplement: Supplementary file 1 [file DataSheet2.pdf]

In statistics, degrees of freedom refer to the number of variables whose values are not restricted when calculating a certain statistic. Degrees of freedom are usually used in sampling distributions. F value is the statistic of F test, which is used to test the variance of two samples. The larger the F value is, the more significant the equation is and the better the fitting degree is. P value is a decreasing indicator of the credibility of the results. The larger the P value is, the more unreliable the result is, and the smaller the P value is, the more reliable the result is. A P value of less than 0.05 was considered statistically significant. In addition, in the statistical interpretation, we generally do not pay attention to the F value, we only need to pay attention to the P value.

**Figure**	**Index**		**Group**	**df**	**F value**	**P value**
Figure 2	B	Infarct volume	Sham vs 0	5	36.571	<0.01
			0 vs 10			>0.05
			0 vs 20			<0.01
			0 vs 40			<0.01
			0 vs Eda			<0.01
	D	Neuronal density level in cortex	Sham vs 0	5	6.985	<0.01
			0 vs 10			>0.05
			0 vs 20			<0.01
			0 vs 40			<0.01
			0 vs Eda			<0.01
		Neuronal density level in CA1	Sham vs 0	5	2.856	<0.01
			0 vs 10			>0.05
			0 vs 20			<0.05
			0 vs 40			<0.05
			0 vs Eda			<0.05
		Nissl bodies density level in cortex	Sham vs 0	5	4.999	<0.01
			0 vs 10			>0.05
			0 vs 20			<0.05
			0 vs 40			<0.05
			0 vs Eda			<0.05
		Nissl bodies density level in CA1	Sham vs 0	5	5.195	<0.01
			0 vs 10			>0.05
			0 vs 20			<0.01

	D		0 vs 40			<0.05
			0 vs Eda			<0.05
	A	Neurological deficit score	1d	9	29.677	>0.05
			7d			>0.05
			14d			<0.05
			21d			<0.05
			28d			<0.05
	B	Use of impaired forelimb	MCAO/R vs MCAO/R+R1 (1 d)	8	13.88	>0.05
			MCAO/R vs MCAO/R+R1 (7 d)			>0.05
			MCAO/R vs MCAO/R+R1 (14 d)			<0.01
			MCAO/R vs MCAO/R+R1 (21 d)			<0.01
			MCAO/R vs MCAO/R+R1 (28 d)			<0.01
	C	Discrimination index	Sham vs MCAO/R	2	4.152	<0.05
			MCAO/R vs MCAO/R+R1			<0.05
	D	Body weight	Sham vs MCAO/R (0d)	8	5.498	>0.05
			MCAO/R vs MCAO/R+R1 (0 d)			>0.05
			Sham vs MCAO/R (7 d)			<0.01
			MCAO/R vs MCAO/R+R1 (7 d)			>0.05
			Sham vs MCAO/R (14 d)			<0.01
			MCAO/R vs MCAO/R+R1 (14 d)			<0.01
			Sham vs MCAO/R (21 d)			<0.01
			MCAO/R vs MCAO/R+R1 (21 d)			<0.05
			Sham vs MCAO/R (28 d)			<0.01
			MCAO/R vs MCAO/R+R1 (28 d)			<0.05
Figure 4	D	DCX$^+$/EdU$^+$ cell	Sham vs MCAO/R	3	32.956	<0.05
			MCAO/R vs MCAO/R+R1			<0.01

			MCAO/R vs MCAO/R+NBP			<0.01
	E	Nestin$^+$/EdU$^+$ cell	Sham vs MCAO/R	3	36.333	>0.05
			MCAO/R vs MCAO/R+R1			<0.01
			MCAO/R vs MCAO/R+NBP			<0.01
	F	NeuN$^+$/EdU$^+$ cell	Sham vs MCAO/R	3	55.732	<0.05
			MCAO/R vs MCAO/R+R1			<0.01
			MCAO/R vs MCAO/R+NBP			<0.01
Figure 5	B	APC$^+$/EdU$^+$ cell	Sham vs MCAO/R	3	56.515	>0.05
			MCAO/R vs MCAO/R+R1			<0.01
			MCAO/R vs MCAO/R+NBP			<0.01
	D	CNPase/β-actin	Sham vs MCAO/R	3	10.998	<0.01
			MCAO/R vs MCAO/R+R1			<0.05
	E	MBP/β-actin	Sham vs MCAO/R	3	2.117	>0.05
			MCAO/R vs MCAO/R+R1			<0.05
	F	Vimentin/β-actin	Sham vs MCAO/R	3	149.582	<0.01
			MCAO/R vs MCAO/R+R1			<0.01
Figure 6	A	Serum BDNF (7 d)	Sham vs MCAO/R	7	25.034	<0.01
			MCAO/R vs MCAO/R+R1			<0.01
		Serum BDNF (28 d)	Sham vs MCAO/R			<0.01
			MCAO/R vs MCAO/R+R1			>0.05
	B	Cortex BDNF (7 d)	Cortex BDNF (7 d)	3	9.313	<0.01
			MCAO/R vs MCAO/R+R1			<0.01
		Cortex BDNF (28 d)	Cortex BDNF (7 d)			<0.01
			MCAO/R vs MCAO/R+R1			>0.05
	C	Serum NGF (7 d)	Sham vs MCAO/R	7	21.426	<0.01
			MCAO/R vs MCAO/R+R1			<0.01
		Serum NGF (28 d)	Sham vs MCAO/R			<0.01

	D		MCAO/R vs MCAO/R+R1			<0.01
	D	Cortex NGF (7 d)	Sham vs MCAO/R	3	3.817	>0.05
			MCAO/R vs MCAO/R+R1			<0.01
		Cortex NGF (28 d)	Sham vs MCAO/R			<0.01
			MCAO/R vs MCAO/R+R1			>0.05
	E	Serum NT-4 (7 d)	Sham vs MCAO/R	7	32.745	>0.05
			MCAO/R vs MCAO/R+R1			<0.01
		Serum NT-4 (28 d)	Sham vs MCAO/R			<0.01
			MCAO/R vs MCAO/R+R1			<0.01
	F	Cortex NT-4 (7 d)	Sham vs MCAO/R	3	5.042	<0.01
			MCAO/R vs MCAO/R+R1			<0.01
		Cortex NT-4 (28 d)	Sham vs MCAO/R			<0.01
			MCAO/R vs MCAO/R+R1			<0.01
	I	SYP/β-actin	Sham vs MCAO/R	3	6.915	>0.05
			MCAO/R vs MCAO/R+R1			<0.01
	J	PSD95/β-actin	Sham vs MCAO/R	3	32.022	<0.01
			MCAO/R vs MCAO/R+R1			<0.05
	K	MAP-2/β-actin	Sham vs MCAO/R	3	14.774	<0.05
			MCAO/R vs MCAO/R+R1			<0.01
	L	Tau-1/β-actin	Sham vs MCAO/R	3	6.592	>0.05
			MCAO/R vs MCAO/R+R1			<0.01
Figure 7	B	BDNF/β-actin (7 d)	Sham vs MCAO/R	3	3.034	<0.05
			MCAO/R vs MCAO/R+R1			<0.05
		BDNF/β-actin (28 d)	Sham vs MCAO/R	3	2.625	>0.05
			MCAO/R vs MCAO/R+R1			<0.05
	C	p-TrkB/TrkB (7 d)	Sham vs MCAO/R	3	42.002	<0.01
			MCAO/R vs MCAO/R+R1			<0.05
		p-TrkB/TrkB (28 d)	Sham vs MCAO/R		18.089	<0.01

	D		MCAO/R vs MCAO/R+R1	3		<0.05
	D	p-CREB/CREB (7 d)	Sham vs MCAO/R	3	5.694	<0.01
			MCAO/R vs MCAO/R+R1			<0.01
		p-CREB/CREB (28 d)	Sham vs MCAO/R	3	47.084	<0.01
			MCAO/R vs MCAO/R+R1			<0.01
	E	p-AKT/AKT (7 d)	Sham vs MCAO/R	3	4.091	<0.01
			MCAO/R vs MCAO/R+R1			<0.05
		p-AKT/AKT (28 d)	Sham vs MCAO/R	3	6.16	<0.01
			MCAO/R vs MCAO/R+R1			<0.01
	I	Glutamate	Sham vs MCAO/R	3	2.624	<0.05
			MCAO/R vs MCAO/R+R1			<0.05
			MCAO/R vs MCAO/R+NBP			>0.05
	J	N-acetyl-L-Aspartate	Sham vs MCAO/R	3	3.323	<0.01
			MCAO/R vs MCAO/R+R1			<0.05
			MCAO/R vs MCAO/R+NBP			>0.05
	K	K^+	Sham vs MCAO/R	3	7.967	<0.01
			MCAO/R vs MCAO/R+R1			<0.01
			MCAO/R vs MCAO/R+NBP			<0.05
Figure 8	C	Proliferation rate	Cont vs 0	5	20.501	<0.01
			0 vs 12.5			
			0 vs 25			<0.01
			0 vs 50			<0.01
			0 vs 100			<0.01
	D	Proliferation rate	Cont vs 0	4	23.680	<0.01
			0 vs 25			<0.01
			25 vs AVA			<0.05
			25 vs LY			<0.01
	F	BDNF/β-actin	Cont vs OGD/R	4	6.478	<0.01
			OGD/R vs R1			<0.01
			R1 vs VA			<0.05
			R1 vs LY			<0.01
	G	p-TrkB/TrkB	Cont vs OGD/R	4	61.23	<0.01

			OGD/R vs R1			<0.01
			R1 vs VA			<0.01
			R1 vs LY			<0.01
	H	p-CREB/CREB	Cont vs OGD/R	4	4.663	<0.01
			OGD/R vs R1			<0.01
			R1 vs VA			<0.05
			R1 vs LY			<0.05
	I	p-AKT/AKT	Cont vs OGD/R	4	5.593	<0.05
			OGD/R vs R1			<0.01
			R1 vs VA			<0.05
			R1 vs LY			<0.01
Figure S1	B	Evans blue leakage	Sham vs MCAO/R	3	22.858	<0.01
			MCAO/R vs MCAO/R+R1			<0.01
			MCAO/R vs MCAO/R+NIM			<0.01
	C	Brain water content	Sham vs MCAO/R	3	38.538	<0.01
			MCAO/R vs MCAO/R+R1			<0.01
			MCAO/R vs MCAO/R+NIM			<0.01
	D	Relative protein expression	Claudin-5 (7 d)	11	325.796	>0.05
			Occludin (7 d)			<0.01
			Zo-1 (7 d)			<0.01
			Claudin-5 (28 d)			<0.01
			Occludin (28 d)			<0.01
			Zo-1 (28 d)			<0.01
