# Supplementary figures and images for: Notoginsenoside R1 Improves Cerebral Ischemia/Reperfusion Injury by Promoting Neurogenesis via the BDNF/Akt/CREB Pathway
Source: Front Pharmacol. 2021 May 7;12:615998. doi: 10.3389/fphar.2021.615998 (PMC8138209; doi:10.3389/fphar.2021.615998)

**Figure 8E**

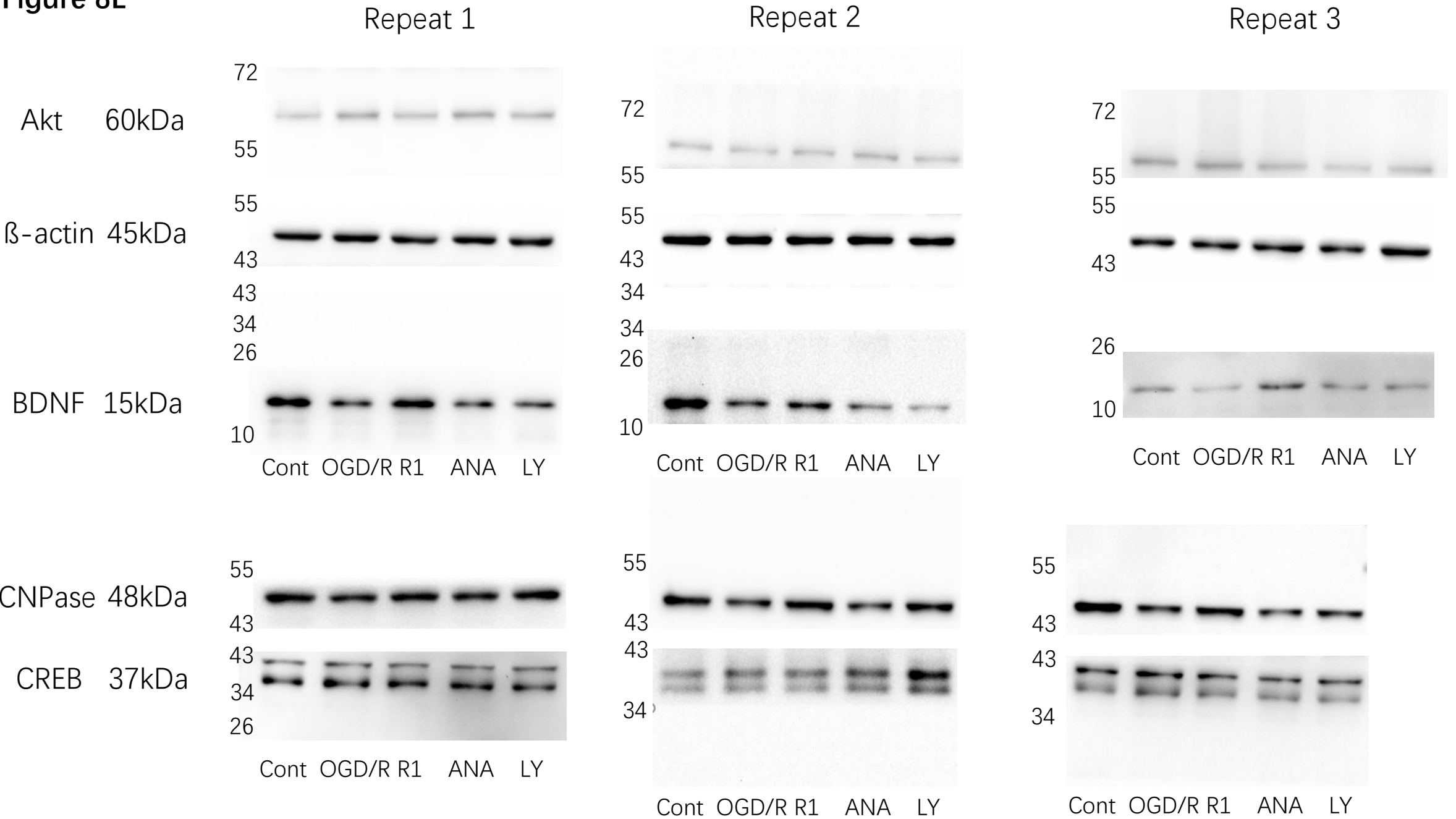

Figure 8E

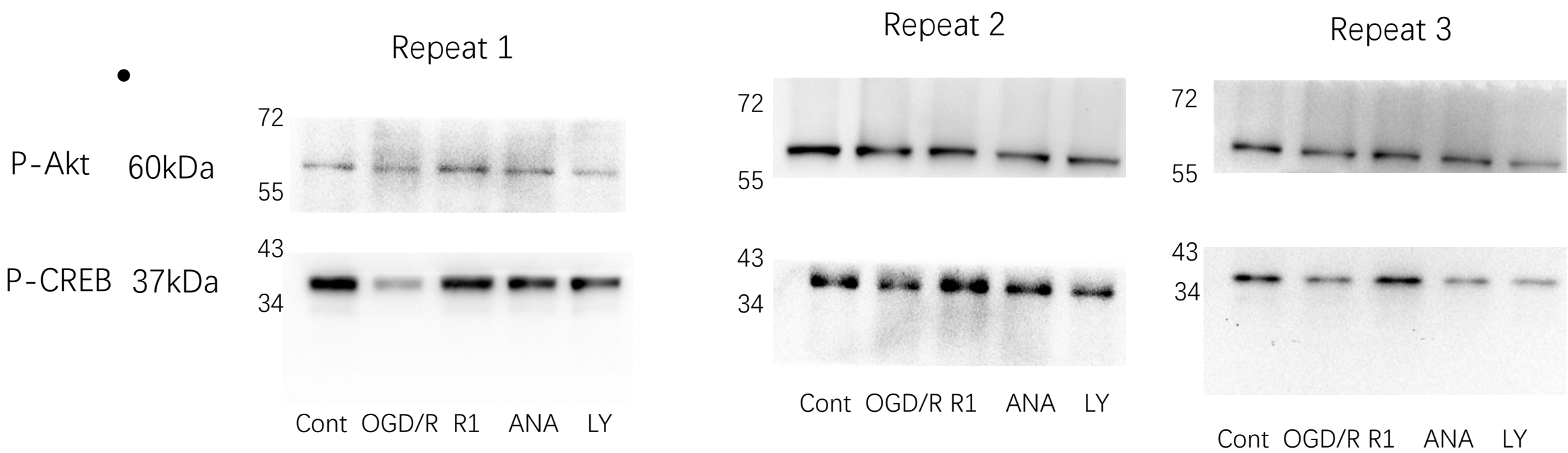

Figure 5, 6

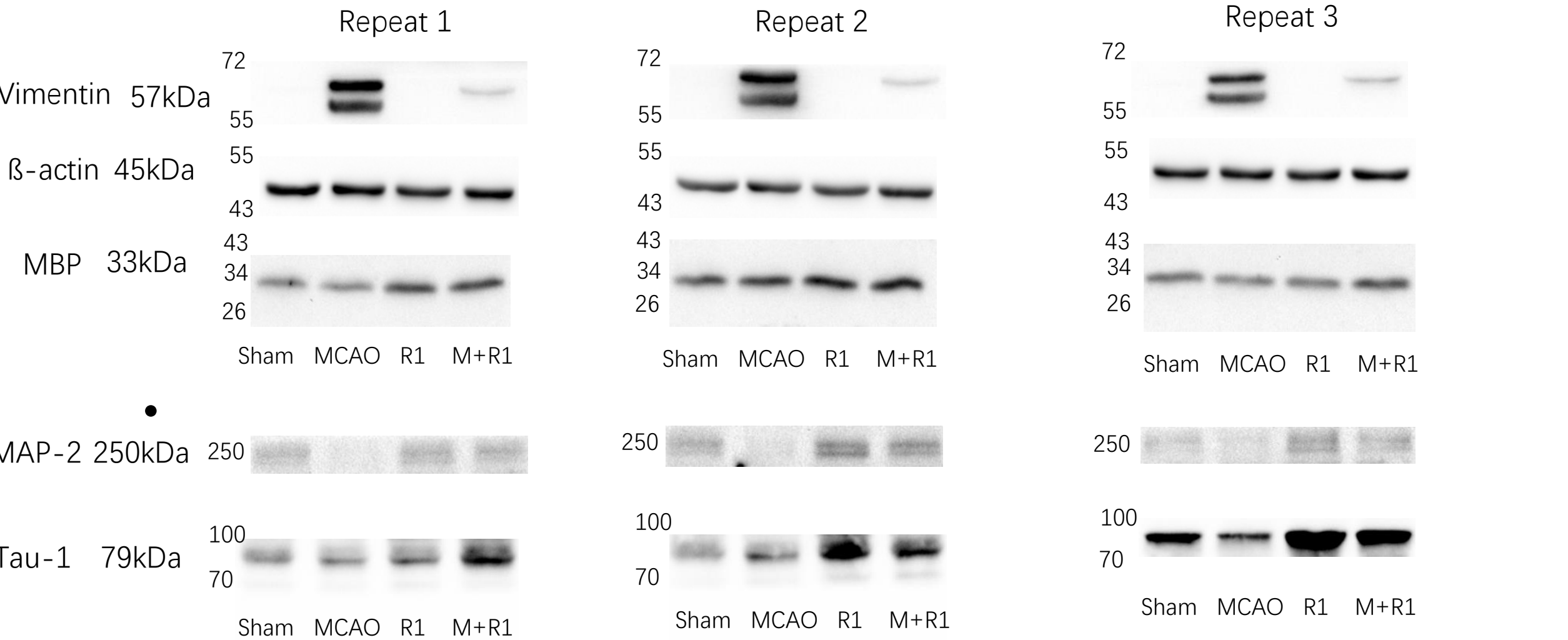

Figure 5, 6

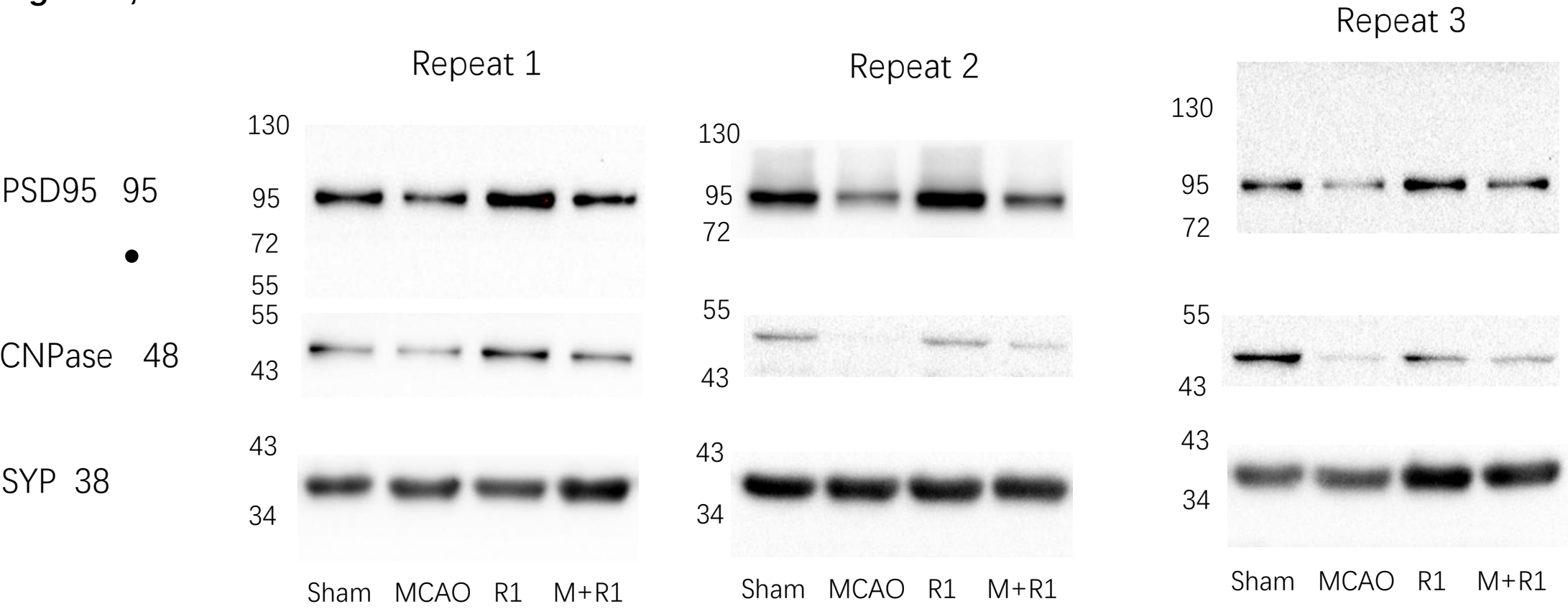

**Figure 7**

**28d**

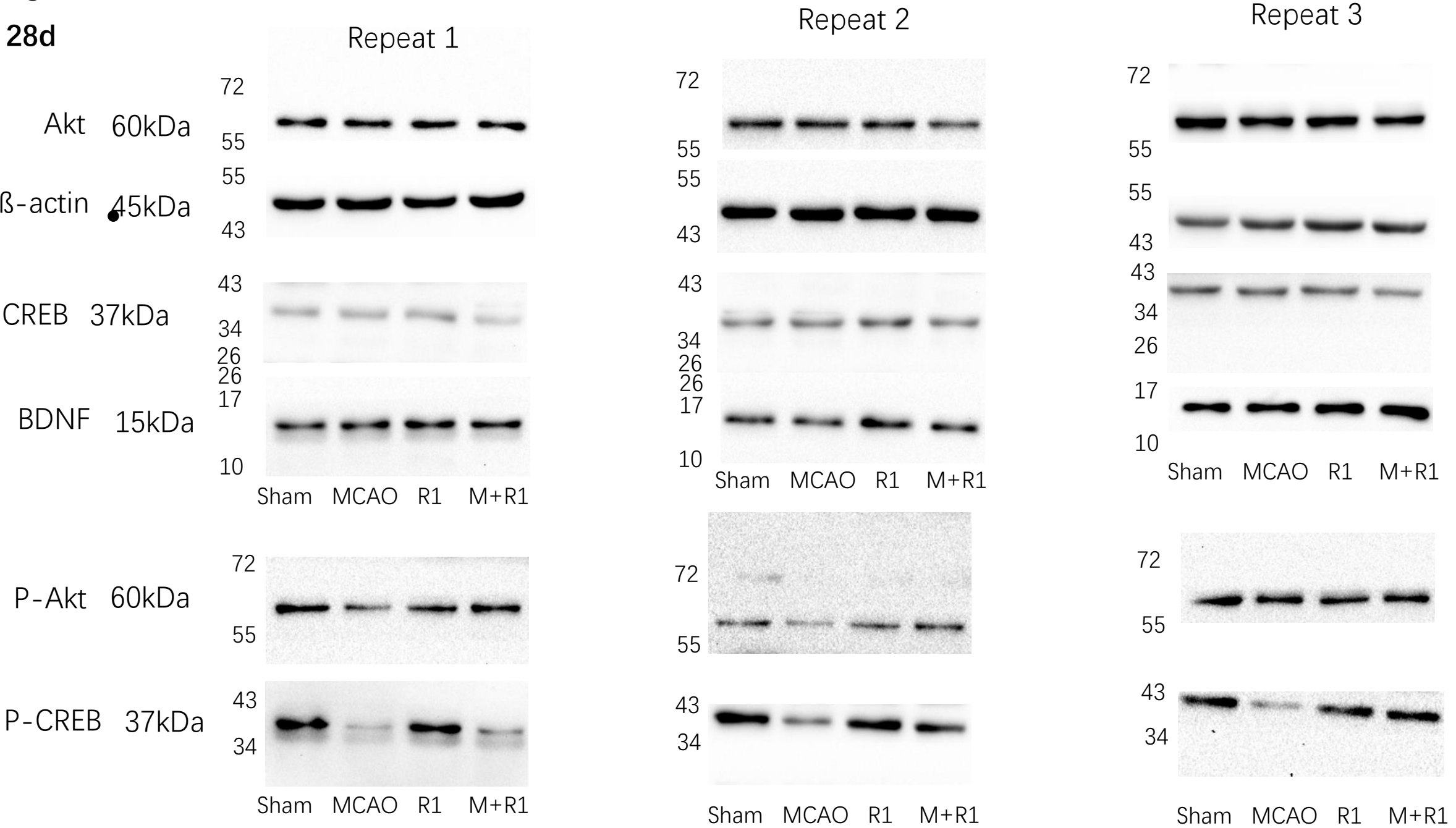

**Figure 7**

**7d**

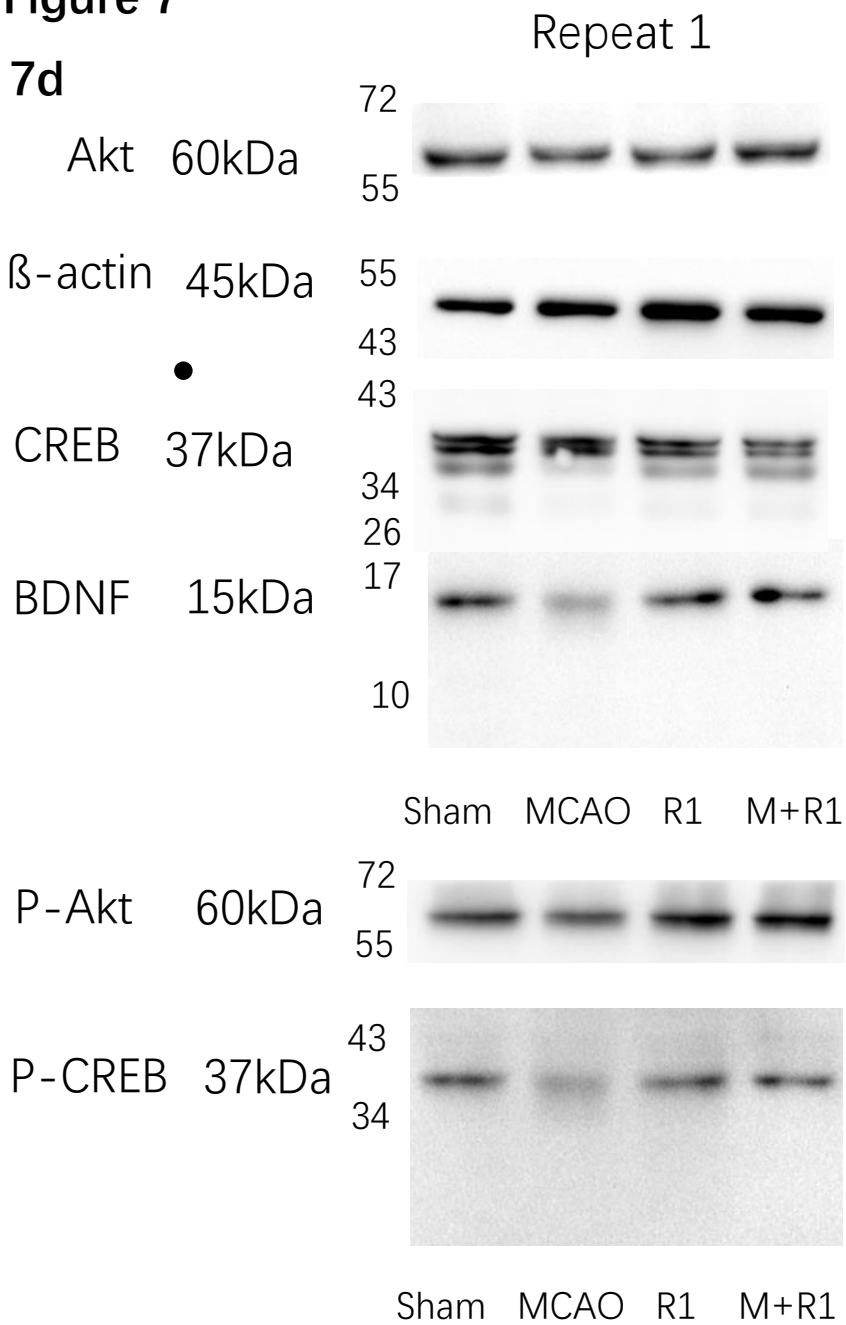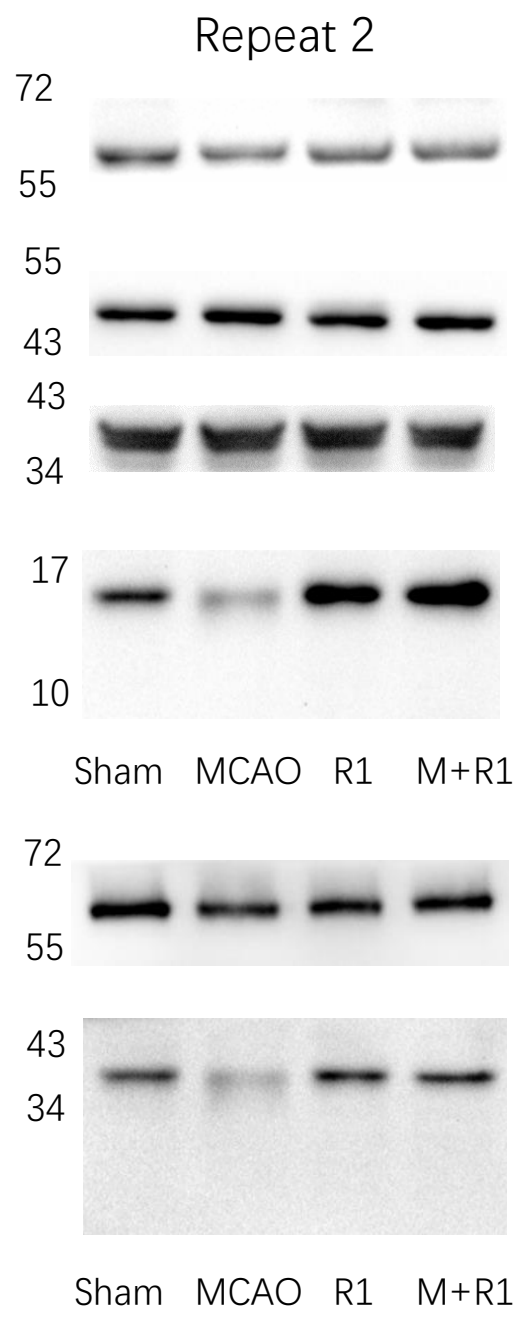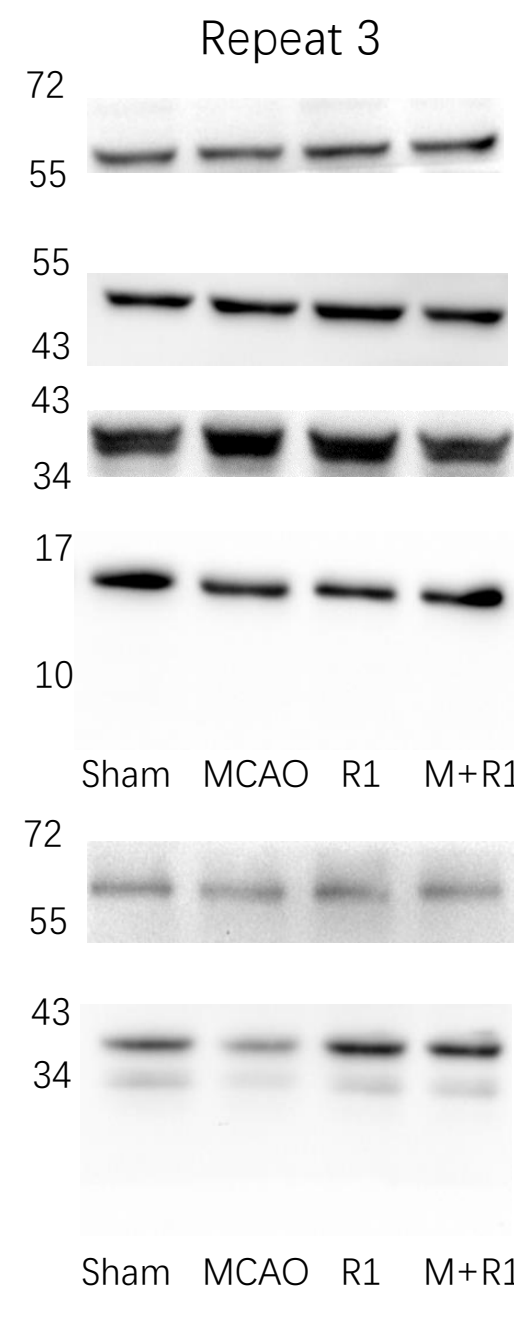

**Figure 7**

**7d**

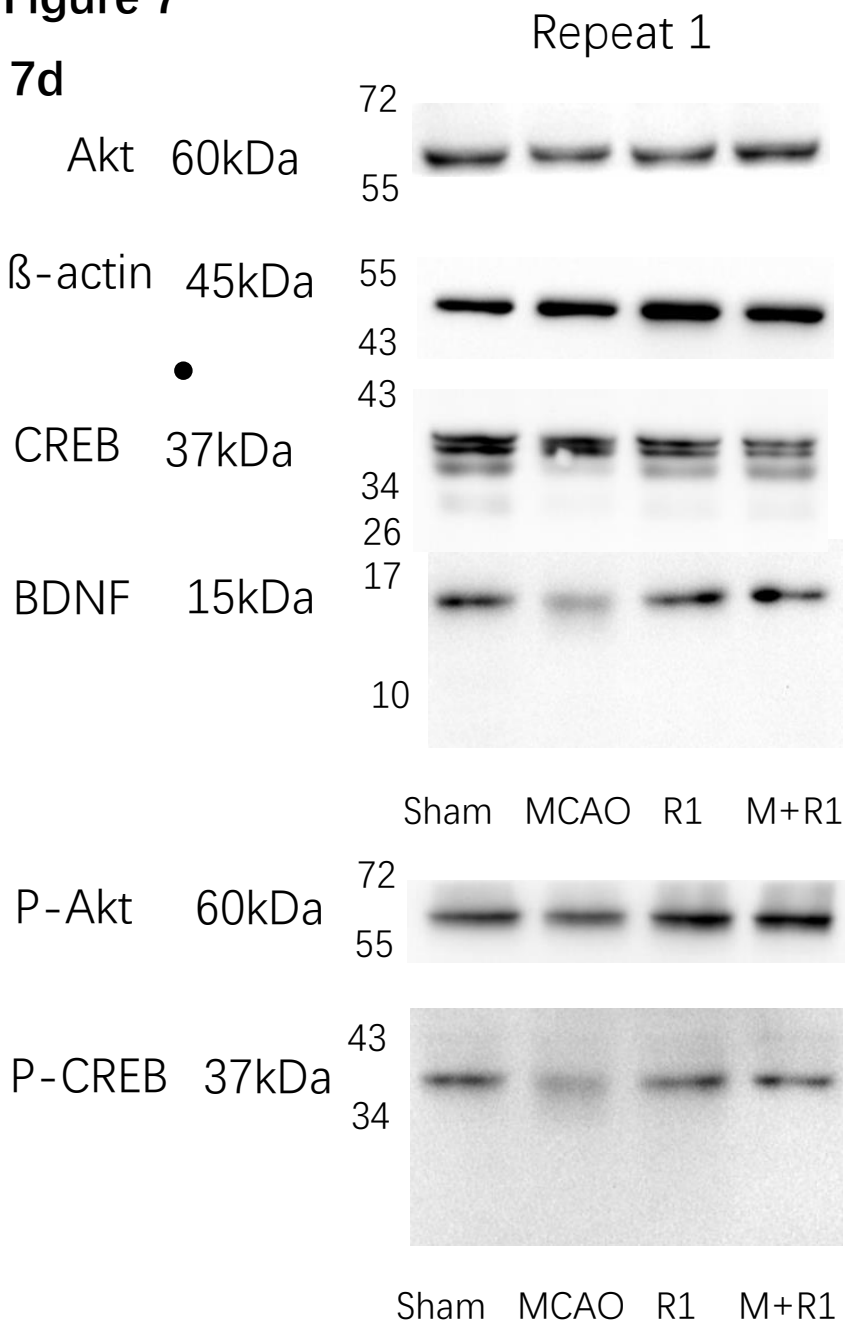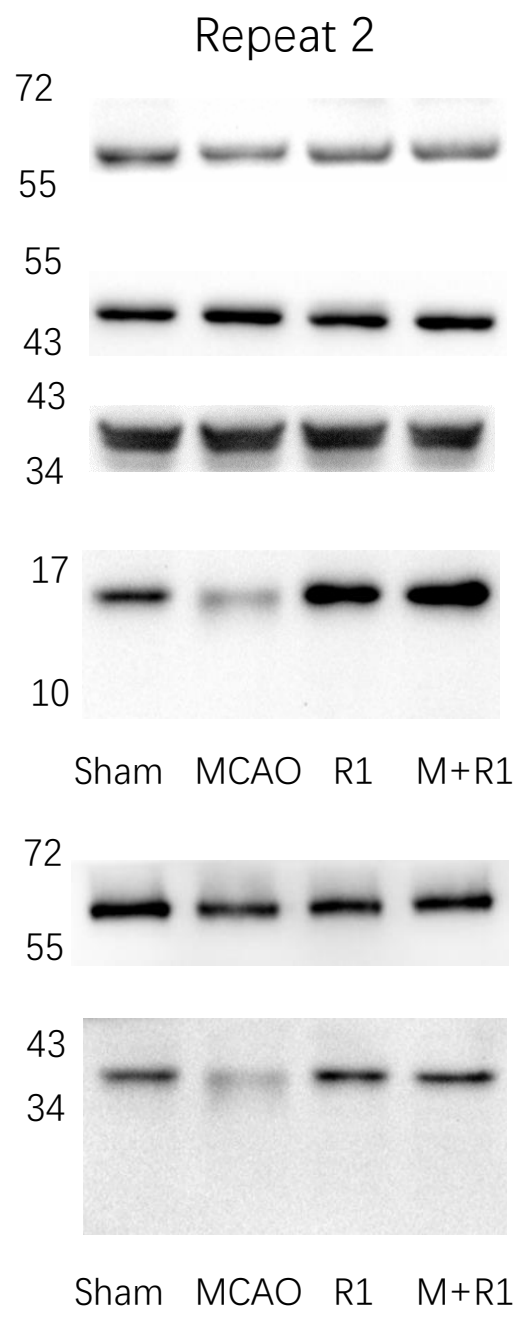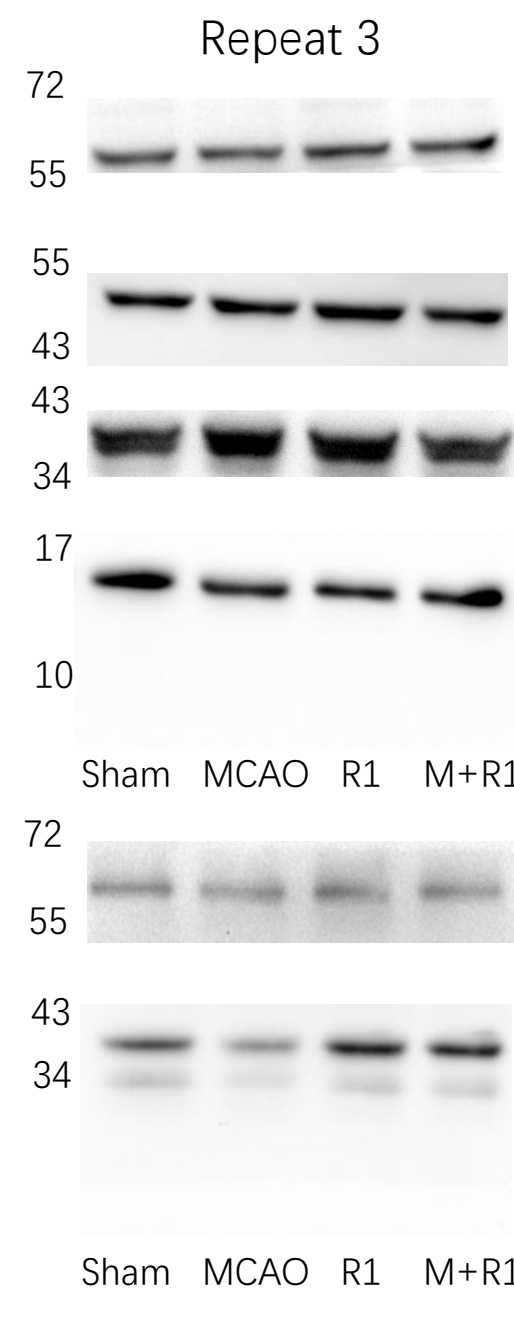

Supplement: Supplementary file 2 [file DataSheet1.PDF]
