# Supplementary material for: Notoginsenoside R1 Improves Cerebral Ischemia/Reperfusion Injury by Promoting Neurogenesis via the BDNF/Akt/CREB Pathway
Source: Front Pharmacol. 2021 May 7;12:615998. doi: 10.3389/fphar.2021.615998 (PMC8138209; doi:10.3389/fphar.2021.615998)
Supplement: Supplementary file 3 [file Image1.pdf]

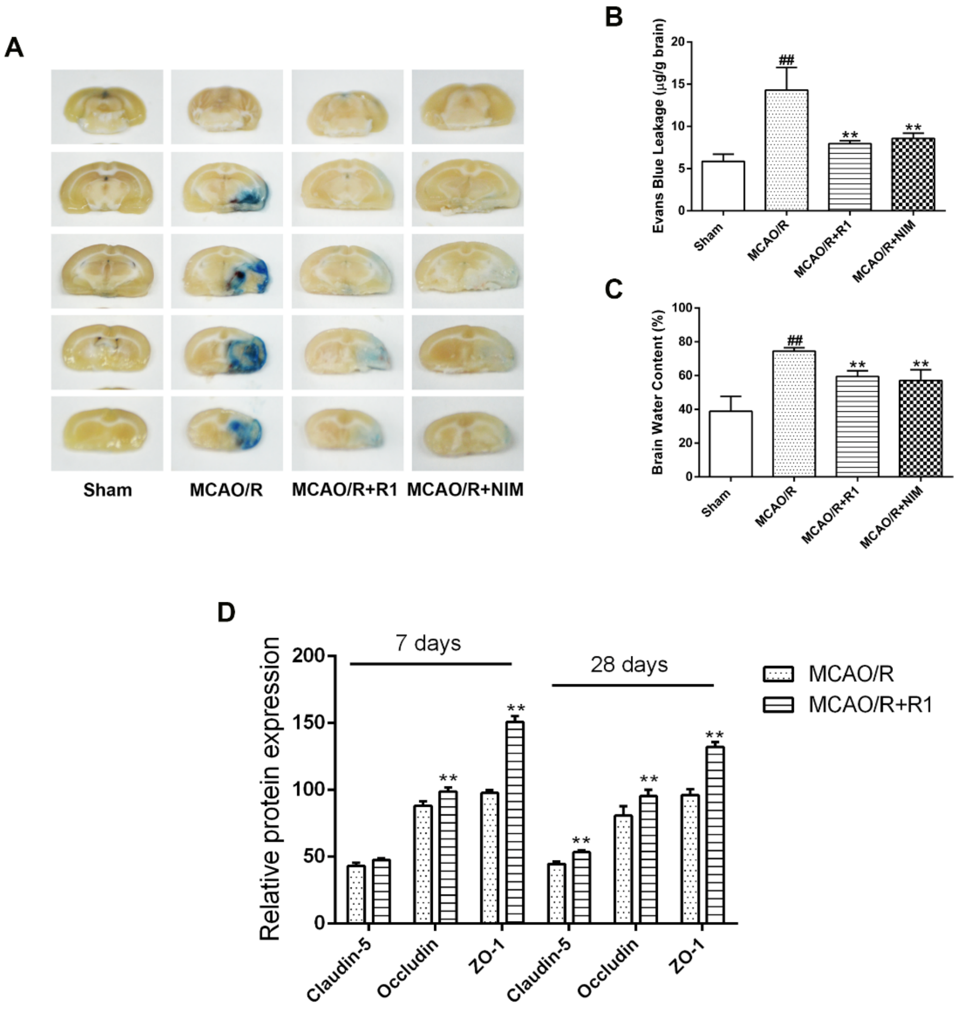

Figure S1. R1 alleviates the BBB disruption. **(A)** Evans blue leakage in ischemia hemispheres of all groups. n = 5 in each group. **(B)** Quantification of the Evans blue leakage in ischemia hemispheres of all groups. n = 5 in each group. **(C)** Brain water content in ischemia hemispheres of all groups. n = 5 in each group. **(D)** Serum levels of BBB-related factors, such as Claudin-5, Occludin and ZO-1, were detected by ELISA on days 7 and 28 after MCAO surgery. n = 5 in each group. Data are expressed as the mean ± SD and were analyzed by ANOVA. ## p<0.01 vs. Sham group; ** p<0.01 vs. MCAO/R group.
